# Understanding the barriers and facilitators of vaccine hesitancy towards the COVID-19 vaccine in healthcare workers and healthcare students worldwide: An Umbrella Review

**DOI:** 10.1371/journal.pone.0280439

**Published:** 2023-04-12

**Authors:** Jemma Louise McCready, Bethany Nichol, Mary Steen, John Unsworth, Dania Comparcini, Marco Tomietto

**Affiliations:** 1 Department of Social Work, Education and Community Wellbeing, Faculty of Health and Life Sciences, Northumbria University, Newcastle upon Tyne, United Kingdom; 2 Department of Social Work, Education and Community Wellbeing, Northumbria University, Newcastle upon Tyne, United Kingdom; 3 Department of Nursing, Midwifery and Health, Faculty of Health and Life Sciences, Northumbria University, Newcastle upon Tyne, United Kingdom; 4 Politecnica delle Marche University of Ancona, Ancona, Italy; 5 Research Unit of Nursing Science and Health Management, University of Oulu, Oulu, Finland; 6 University of Bari “Aldo Moro”, Bari, Italy; Universitas Syiah Kuala, INDONESIA

## Abstract

**Background:**

Healthcare workers (HCWs) and healthcare students display high levels of vaccine hesitancy with impact on healthcare provision, patient safety, and health promotion. The factors related to vaccine hesitancy have been reported in several systematic reviews. However, this evidence needs to be synthesised, as interventions to reduce vaccination hesitancy in this population are needed.

**Methods:**

This Umbrella Review aimed to explore the barriers and facilitators of vaccine hesitancy toward the COVID-19 vaccine for HCWs and healthcare students. The review was performed and reported in accordance with Joanna Briggs Institutes guidelines and the Preferred Reporting Items for Systematic Reviews and Meta-analysis (PRISMA) guidelines. A protocol was preregistered on PROSPERO (CRD42022327354). Eight databases were searched from November 2019 to 23^rd^ May 2022 to identify any systematic reviews that explored factors associated with hesitancy towards the COVID-19 vaccine for HCWs or healthcare students.

**Results:**

A total of 31 studies were included in the review. The majority of studies (71%) were appraised as strong or moderate quality and there was a slight degree of overlap (<5%) of primary studies between the reviews. Vaccine hesitancy was more common among HCWs and healthcare students in specific occupational roles (e.g. nurses) than others (e.g. physicians). Frequent reasons for hesitancy were related to sociodemographic factors (gender, age, ethnicity), occupational factors (COVID-19 exposure, perceived risk, mandatory vaccination), health factors (vaccination history), vaccine-related factors (concerns about safety, efficacy, side-effects, rapid development, testing, approval and distribution of the vaccine), social factors (social pressure, altruism and collective responsibility), distrust factors (key social actors, pandemic management), information factors (inadequate information and sources, exposure to misinformation).

**Conclusion:**

The results from this Umbrella Review have wide-reaching implications for the research area, healthcare systems and institutions and governments worldwide. Designing tailored strategies for specific occupational groups is pivotal to increasing vaccine uptake and securing a safe healthcare provision worldwide.

## 1. Introduction

A delay or refusal to get vaccinated despite the availability of vaccines, also known as vaccine hesitancy, is considered one of the top ten threats to global health by the World Health Organization (WHO) [1]. Reasons for hesitancy are complex and are driven by both an individual’s perceptions and attitudes towards a particular vaccine or vaccines in general, their sociodemographic characteristics (e.g. gender, ethnicity) and contextual factors (e.g. trust in experts, perceived risk) [2]. The indecision and uncertainty about vaccination are amenable to change should emerging concerns be addressed adequately [3]. The importance of understanding the factors driving vaccine hesitancy has become increasingly apparent throughout the current global outbreak of coronavirus disease 2019 (COVID-19). As COVID-19 spread around the globe and the number of active infections and death tolls increased, the need for a vaccine to fight the pandemic became increasingly more critical. However, the rapidity of vaccine development and approval and concerns regarding the vaccine’s safety, effectiveness and side effects have contributed to a lack of vaccine confidence and lower vaccination uptake rates in the general population [4].

Research has identified that healthcare workers (HCWs) and healthcare students are one such sub-group which are displaying considerable hesitancy towards accepting a COVID-19 vaccine. For example, a review exploring the prevalence of hesitancy in more than 75,000 HCWs worldwide found that a fifth of professionals were reluctant to accept a COVID-19 vaccine [5]. For healthcare students, rates of hesitancy were found to mimic those of practising professionals, whereby nearly a fifth of healthcare students (~20,000 students across 39 countries) were also hesitant to accept a COVID-19 vaccination [6]. The high rates of hesitancy in this population are of significant concern for several reasons. Firstly, HCWs and healthcare students are at an increased risk of contracting the virus, which would result in greater staff absence at a time when healthcare is in greater demand [7]. Secondly, HCWs and healthcare students are more likely to be a vector of the virus, transmitting infections to clinically vulnerable patients, the elderly, family members and colleagues, increasing active infections [8]. Furthermore, vaccination recommendations from HCWs have been shown to increase vaccine acceptance in the general population [9, 10]. Therefore, understanding the factors contributing to vaccine hesitancy and implementing effective strategies to tackle this problem is pivotal in reducing the transmission of COVID-19, increasing vaccination uptake in the general population, and maintaining a safe healthcare provision. Over the last 18 months, there has been a rapid growth of primary studies exploring factors associated with COVID-19 vaccine hesitancy for this population in various countries worldwide. As a result, numerous systematic reviews and meta-analyses have also been conducted between the years 2020 and 2022. However, these reviews failed to provide conclusive and univocal evidence on the COVID-19 vaccine acceptance rate, by reporting a variation from 41.84% [29] to 80.41% [45] in HCWs and from 53.85% [29] to 82.37% [35] in healthcare students. Moreover, when systematic reviews and meta-analyses on the same topic are conducted and published in the same years, there is a greater risk of inconclusive results, which, therefore, may have consequences for the clinical decision-making process at both individual and organizational levels. As intervention strategies depend upon this evidence, despite the several reviews published on the topic, there is a need to compile evidence from previously published systematic reviews and meta-analyses to provide an overview of the topic. Therefore, an Umbrella Review will be undertaken to synthesize evidence on the factors associated with hesitancy and acceptance of the COVID-19 vaccine for HCWs and healthcare students. Additionally, suggestions to address the identified drivers will be discussed.

## 2. Methods

A systematic literature search was performed and reported in accordance with the Joanna Briggs Institutes (JBI) guidelines for Umbrella Reviews [11]. The Preferred Reporting Items for Systematic Reviews and Meta-analysis (PRISMA) guidelines were utilised to report the results [12]. A protocol was registered with PROSPERO prior to the commencement of the review (registration no: CRD42022327354). The search strategy and syntax were peer-reviewed by an expert librarian using the Peer Review of Electronic Search Strategies (PRESS) checklist [13] and modifications were implemented prior to the commencement of searches.

### 2.1 Eligibility criteria

The inclusion and exclusion criteria are outlined in Table 1. Studies were eligible for inclusion if they: (i) conducted a systematic review (with or without meta-analysis); (ii) included primary sources utilising a quantitative methodology; (iii) investigated factors related to COVID-19 vaccine hesitancy or vaccine uptake; (iv) included a population or subpopulation of HCWs or healthcare students aged between 18–65 years; (v) were published after 2019 (due to the nature of the topic); (vi) and published in the English language. This review will include peer-reviewed and pre-printed material due to the fast nature of the research surrounding the COVID-19 pandemic. Other non-peer-reviewed material will be excluded (e.g. letters to editors, opinion pieces, commentaries).

**Table 1:** Inclusion and exclusion criteria for eligibility in the review.

	Inclusion criteria	Exclusion criteria
Participants/population	Adults aged 18–65	Children, adolescents or older adults/elderly populations (>65)
	Healthcare workers (any roles) and healthcare students	Populations not working in a healthcare setting
	Male and females	No exclusion criteria
	Human studies	Animal studies
Intervention	COVID-19 vaccine	All other vaccines (e.g. influenza)
Comparator/control	Any group(s) or control group(s)	No exclusion criteria
Outcomes	Any outcomes that serve as a determinant of vaccine hesitancy whether as either a barrier or a facilitator	Studies that only assess levels or prevalence of vaccine hesitancy without a discussion of the determinants of vaccine hesitancy
Setting	Any geographical location and any cultural factors (e.g. race/ethnicity, gender)	No exclusion criteria
Study type	Systematic reviews, with or without meta-analyses containing primary quantitative observational studies (e.g. cross-sectional)	Theoretical studies or text and opinion as their primary source of evidence
	Quantitative data	Qualitative and mixed-methods data
Additional criteria	Peer-reviewed or pre-printed studies	Non-peer-reviewed material
	Must be published in English	Studies published in any language other than English
	Published after 2019	Published prior to 2019

### 2.2 Information sources and search strategy

A systematic search was conducted on the following databases: CINAHL, Cochrane Library, PubMed, ProQuest (COVID-19 Database and International Bibliography of Social Sciences (IBSS)), ScienceDirect, Web of Science, Google Scholar and Epistemonikos. Databases were searched from November 2019 up to 23^rd^ May 2022. The search syntax (Table 2) was entered into the databases as free text rather than MeSH terms. Boolean operators and truncation were utilised if supported by the database. The reference lists of included papers were hand-searched to identify citations not retrieved in the initial searches.

**Table 2:** Search syntax used to search databases.

(COVID-19) OR (COVID19) OR (COVID 19) OR (SARS-CoV-2) OR (SARS-CoV2) OR (SARSCoV2) OR (SARSCoV-2) OR (SARS coronavirus 2) OR (2019 nCoV) OR (2019nCoV) OR (2019-novel CoV) OR (nCov 2019) OR (nCov 19) OR (severe acute respiratory syndrome coronavirus 2) OR (novel coronavirus disease) OR (novel corona virus disease) OR (corona virus disease 2019) OR (coronavirus disease 2019) OR (novel coronavirus pneumonia) OR (novel corona virus pneumonia) OR (severe acute respiratory syndrome coronavirus 2) OR (covid-19 innoculat*) OR (covid-19 immuni?*)	AND	(vaccine hesitan*) OR (vaccine accept*) OR (vaccine refus*) OR (vaccine reluct*)	AND	(healthcare worker) OR (health professional) OR (health personnel) OR (medical staff) OR (medical student*) OR (healthcare student*) (doctor*) OR (nurs*) OR (student nurs*)

### 2.3 Selection of sources of evidence

All retrieved citations were imported into EndNote and duplicates were removed per the de-duplication process proposed by Bramer and colleagues [14]. The remaining articles were exported to an EndNote-generated XML file format and uploaded into Rayyan, which was used to facilitate screening processes [15]. The titles and abstracts of all retrieved citations were independently screened for eligibility against the inclusion criteria (JM). A random allocation of 10% of citations was screened by a second reviewer (BN). Inter-rater agreement, assessed using Cohen’s Kappa (***κ*)**, was considered to be *‘good’* (***κ*** = 0.61; 95% CI 0.48–0.74) [16]. Full-texts for the eligible citations were retrieved and independently screened for relevance to the review aim (JM). A second reviewer (BN) independently screened a random allocation (25%) of full-texts for eligibility. The inter-rater agreement for full-text screening was considered to be *‘moderate’* (***κ*** = 0.41; 95% CI 0.07–0.76) [16]. Disagreements were resolved by a third reviewer (MT). In this study, the inter-rater agreement was satisfactory. For this reason and according to the guidelines [16], the random allocation of 10% of citations has been considered adequate for screening.

### 2.4 Assessment of methodological quality

Methodological quality of the reviews was assessed using the JBI Critical Appraisal Checklist for Systematic Reviews and Research Syntheses [17]. The 11-question checklist was used to assess the possibility of bias in three areas of the review: 1) design (i.e. explicitness of review questions, appropriateness of inclusion criteria and search strategy, adequacy of search sources and appropriateness of critical appraisal tool); 2) conduct (i.e. minimisation of bias during critical appraisal processes and data extraction and assessment of publication bias); and 3) analysis (i.e. appropriateness of synthesis, support for policy recommendations and appropriateness of research directives) [17]. The quality ranking framework, devised by Kilich and colleagues [18], was used to score and interpret the quality of the reviews. The 4-item response scale was scored as follows: ‘*yes*’ = 2 points, ‘*no*’ = -2 points, ‘*unclear*’ = -1 point and ‘*not applicable*’ = 0 points. Scores were summed to derive a total score which was used to categorise the review as either ’*very low quality*’ (<0), ’*low quality*’ (0 ≤—< 5), ’*moderate quality*’ (5 ≤—< 10) or ’*strong quality*’ (≥ 10) [18]. As the aim of an umbrella review is to provide a comprehensive overview of the literature [11, 17], results from the quality assessment were not used to define inclusion or exclusion as the methodological standards for a systematic or a scoping review were assured for each paper included. Accordingly, the inclusion and exclusion criteria focused on the research aim and questions of this umbrella review [17]. One reviewer (JM) conducted the critical appraisal assessment in full and a random allocation of 10% of the reviews was critically appraised by the second reviewer (BN). Both reviewers conducted the quality assessment independently and discrepancies were resolved by a third reviewer (MT).

### 2.5 Assessment of overlap

The degree to which the included reviews contained the same primary studies was assessed using the Corrected Covered Area (CCA) method recommended by Pieper and colleagues [19]. An excel sheet was created which cross-linked the individual reviews with all of the primary publications included in the reviews. Tick marks were used to indicate which reviews included which primary studies. Only the primary studies relating to HCWs, or healthcare students were included in the assessment, as including primary studies irrelevant to the review aim (e.g. for other populations) would result in an inaccurate estimation of the degree of overlap. The total number of primary studies (N), total number of unique primary studies (R) and the number of reviews included (C) were used to calculate the CCA ((N–R)/((RxC)–R)). The CCA score was interpreted as follows: 0–5 = ‘*Slight overlap*’; 6–10 = ‘*Moderate overlap*’; 11–15 = ‘*High overlap*’; and >15 = ‘*Very high overlap* [19].

### 2.6 Data collection

The JBI Data Extraction Form for Review of Systematic Reviews and Research Syntheses [11] was used to capture the following data: study details (author/year, objectives, participants (characteristics/total number), setting/context, description of interventions/phenomena of interest), Search details (sources searched, search date range, date range of included studies), descriptive details of the included studies (number of studies, types of studies, country of origin), Critical appraisal details (appraisal instruments used, appraisal rating) and Analysis (method of analysis, outcome assessed, results/findings, significance/direction, heterogeneity).

One reviewer (JM) independently extracted the required data from the included articles into the data extraction form created in Excel. Another research team member (BN) independently extracted data from a random subset (10%) of the included articles. Both data extraction forms were cross-checked for accuracy and discrepancies were resolved by a third reviewer (MT).

### 2.7 Data summary

Firstly, tabular summaries describing the characteristics of the included reviews and the sociodemographic characteristics of the participants were created. Secondly, the results from the reviews were thematically analysed to identify patterns across the evidence base. The determinants of vaccine hesitancy and acceptance were organised into categories (e.g. sociodemographic factors, vaccine-specific factors) and narrative summaries for each population group were produced.

## 3. Results

### 3.1 Selection of included studies

**Fig 1:**
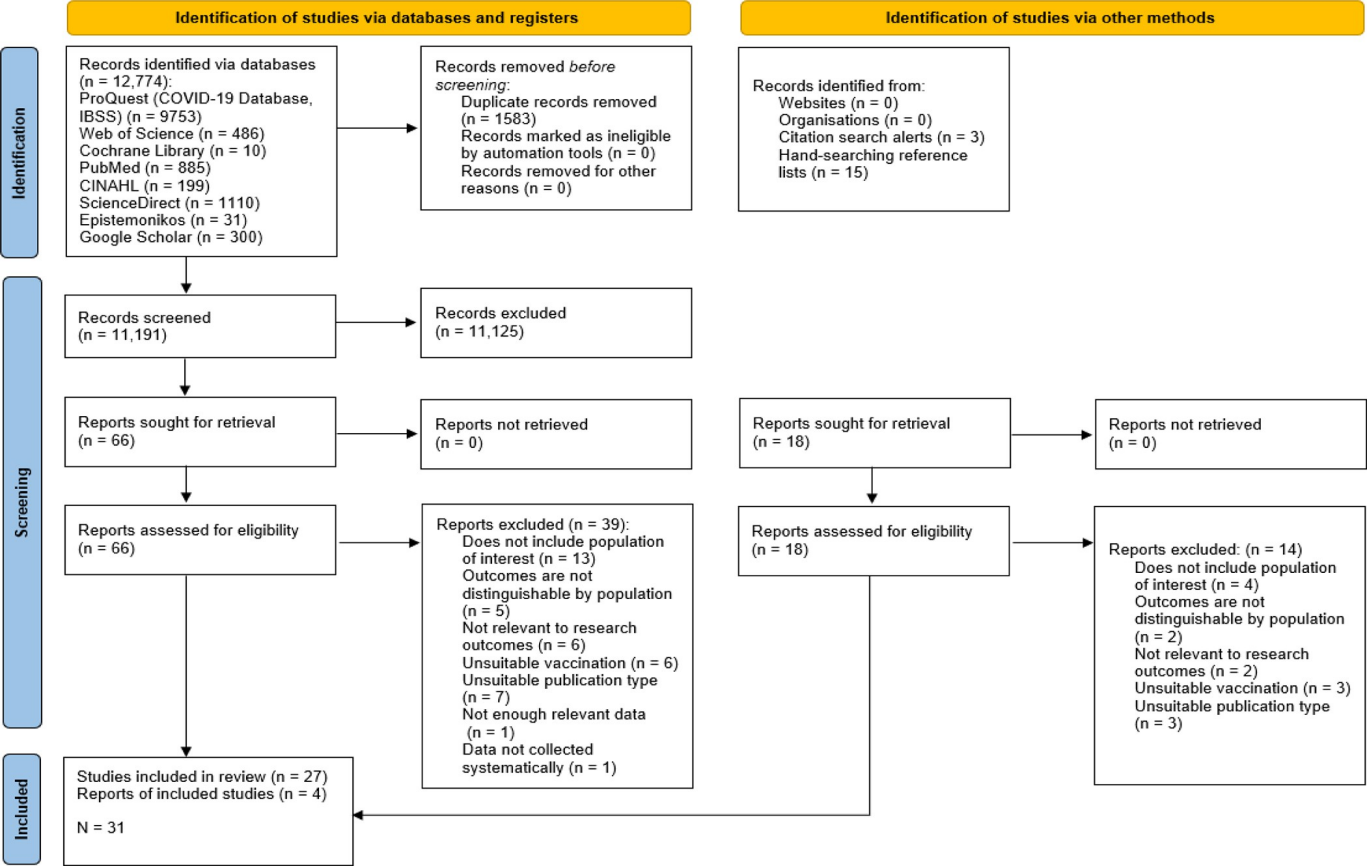
PRISMA flowchart [12] detailing the identification and selection of research syntheses for inclusion in the Umbrella Review.

The search strategy generated 12,774 citations, of which 11,191 were unique. Following title and abstract screening, 11,125 citations were eliminated as they did not meet the initial inclusion criteria. Full-text screening was conducted on 66 papers and 39 were excluded. Main reasons for exclusion were that the review did not include populations of interest (e.g. HCWs or healthcare students), vaccination of interest (e.g. COVID-19), outcomes of interest (e.g. barriers of facilitators of vaccine hesitancy) or were of an unsuitable publication type (e.g. letters to editors, commentaries). Hand-searching the reference lists of the 27 eligible reviews identified 15 citations of interest; however, after full-text screening, only 1 citation was eligible for inclusion. Monitoring of database search alerts for 30 consecutive days identified an additional three eligible articles. A total of 31 reviews met the inclusion criteria and were included (Fig 1).

### 3.2 Characteristics of included studies

Characteristics of the included reviews are outlined in Table 3. Of the 31 reviews included, 27 were published as peer-reviewed journal articles, three were published on pre-print servers [20–22] and one was a peer-reviewed pre-proof [23]. There were eight systematic reviews and meta-analyses [24–31], 16 systematic reviews [20, 21, 32–40] (five categorised as rapid [41–45]), six scoping reviews [5, 22, 46–49] and one integrative review [23]. All reviews included quantitative, cross-sectional primary studies, while three included longitudinal study designs [32, 35, 45] and one included mixed-method surveys [45]. Nineteen reviews were published in 2021 and 12 in 2022, which captured primary studies published from February 2020 to August 2021 [31]. Nine reviews were published in Asia (China [26, 28, 42], Jordan [32], Thailand [33], Malaysia [27], Bangladesh [29], Pakistan [40], Iran [41]), eight in the USA [5, 23, 30, 36, 43, 45, 47, 48], seven in Europe (Italy [35, 38], The Netherlands [22, 49], Greece [25], Czech Republic [37], Slovakia [31]), six in Africa (Ethiopia [21, 34, 39], Ghana [24], Nigeria [44], Burkina Faso [20]) and one in Canada [46]. Of the 31 reviews, 14 assessed the methodological quality of the primary studies included in their review. Commonly used appraisal tools were the JBI critical appraisal tool for cross-sectional studies [25, 27, 29, 30, 42] and the Newcastle-Ottawa scale (NOS) for cross-sectional studies [21, 24, 26, 39]. Where used, a large proportion of the primary studies were assessed as ‘*high to moderate quality*’ (JBI tool) or as ‘*low to moderate risk of bias*’ (NOS tool).

**Table 3:** Main characteristics of studies included in the Umbrella Review.

No.	Author, year [reference]	Country	Type of review	Sources searched; Search period	No of studies included	Date range of included studies (earliest date—latest date)	Country of origin of included studies	Quality Assessment Tool and Results
1	Ackah et al., 2021 [24]	Ghana	Systematic review and meta-analysis	MedLine/PubMed, Google Scholar, Africa Journal Online; January 2020—September 2021	N = 21. **HCWs**: n = 21	March 2020—June 2021	North Africa, West Africa, East Africa, Central Africa, Southern Africa	NOS for cross-sectional studies Low risk of bias (7–10): n = 12; Moderate risk of bias (5–6): n = 8; High risk of bias (0–4): n = 1
2	Al-Amer et al., 2021 [32]	Jordan	Systematic review	CINAHL, Cochrane Library, Google Scholar, ProQuest, PsycINFO, PubMed, Scopus; Up to 31^st^ December 2020	N = 30. **HCWs**: n = 9	**HCWs:** 2020	**HCWs**: Italy, Israel, France, Malta, Germany, Indonesia, USA, Republic of Congo, China	JBI for Quasi-experimental design High quality (>80%): n = 16; Moderate quality (79%-60%): n = 10; Poor quality (59%-30%): n = 3
3	AlShurman et al., 2021 [46]	Canada	Scoping review	Scopus, PubMed, CINAHL, PsycINFO; November 2019 -December 2020	N = 48. **HCWs:** n = 8 **University students/ academics:** n = 4	**HCWs:** 2020 **University students and academics:** 2020	**HCWs**: Democratic Republic of the Congo, China, France, Germany, Malta, Turkey **University students and academics:** Italy, Malta, Germany, France	NR
4	Biswas et al., 2021 [5]	USA	Scoping review	PubMed, EBSCO Host, pre-print servers (medRxiv), Google Scholar; February 2020—February 2021	N = 35. **HCWs**: n = 35	February 2020—January 2021	USA, France, Saudi Arabia, China, Congo, Malta, Greece, Italy, Canada, Israel, Indonesia, Iran, Turkey, Egypt, Poland	NR
5	Caiazzo & Stimpfel, 2022 [23]	USA	Integrative review	CINAHL (via EBSCO), Medline (via PubMed), Web of Science, PsychInfo; Up to July 2021	N = 18. **HCWs**: n = 18	August 2020—March 2021	USA: South, Northeast, West, Midwest, Mid-Atlantic, Multiple US regions	NR
6	Dadras et al., 2022 [33]	Thailand	Systematic review	PubMed, Scopus, Web of Science, Cochrane Library; Up to 5^th^ August 2021	N = 45. **HCWs**: n = 9	**HCWs:** NR	**HCWs:** Saudi Arabia, Israel, Qatar, Egypt, Palestine	NR
7	Fattah et al., 2022 [41]	Iran	Rapid systematic review	MedLine, EMBASE, Scopus, Web of Science, Cochrane Library, CIVILICA, Google Scholar; Up to November 2021	N = 37. **HCWs:** n = 2	**HCWs:** NR	**HCWs:** Israel, USA	NR
8	Galanis et al., 2021 [25]	Greece	Systematic review and meta-analysis	PubMed, MEDLINE, Scopus, Web of Science, ProQuest, CINAHL, pre-print services (medRxiv); 1^st^ January 2021 - 14^th^ July 2021	N = 24. **HCWs**: n = 24	February 2020—March 2021	Asia (China, Turkey, Kingdom of Saudi Arabia, Vietnam and Kuwait), North America (USA and Canada), Europe (France, Germany and Greece), Africa (Democratic Republic of Congo, Eastern Cape and Zambia), Multicentre: France, Belgium and Canada	JBI for cross-sectional studies Poor quality (≤3 points): n = 0; Moderate quality (4–6 points): n = 6; Good quality (7–8 points): n = 18
9	Geng et al., 2022 [26]	China	Systematic review and meta-analysis	Medline/PubMed, Cochrane Library, Web of Science, China National Knowledge Infrastructure (CNKI); Up to 31^st^ December 2021	N = 34. **Medical students:** n = 15 **Non-medical students:** n = 18 **Medical and non-medical students:** n = 1	**Medical students:** June 2020—March 2021 **Non-medical students:** March 2020—April 2021 **Medical and non-medical students:** December 2020	**Medical students:** USA, China, Kuwait, Israel, Malta, Egypt, India, Uganda, Slovakia, Multicentre: Greece, Albania, Cyprus, Spain, Italy, Czech Republic and Kosovo, Multicentre: Albania, Canada, Croatia, Ecuador, Estonia, Indonesia, Iran, Iraq, Italy, Latvia, Lebanon, Lithuania, Malaysia, Nepal, Pakistan, Palestine, Portugal, Russia, Sudan, Tunisia, Turkey and USA; Slovenia, Poland and Serbia **Non-medical students:** USA, China, Italy, Indonesia, Malaysia, Jordan, France, United Arab Emirates, Bangladesh **Medical and non-medical students:** Poland	NOS for cross-sectional studies High quality (≥6): n = 24; Moderate quality (5–4): n = 10; Low quality (≤3): n = 0
10	Hajure et al., 2021 [34]	Ethiopia	Systematic review	PubMed, Science Direct, Google Scholar; Up to 20th July 2021	N = 24. **HCWs:** n = 24	March 2020–2021	USA Saudi Arabia, Ghana, Italy, France, Greece, Congo, Poland, Romania, Taiwan, Canada, Colombia, Slovenia, the United Arab Emirates, Arabic-speaking countries, Pakistan, Nepal, Belgium, Egypt, Israel	National Institute of Health quality assessment tool for observational cohort and cross-sectional studies Good: n = 15; Fair: n = 6; Poor: n = 3
11	Joshi et al., 2021 [47]	USA	Scoping review	PubMed; Up to 15^th^ December 2020	N = 22. **HCWs:** n = 3	**HCWs:** February 2020—July 2020	**HCWs:** China, Israel, France	NR
12	Khubchandani et al., 2022 [48]	USA	Scoping review	PubMed, EBSCO Host, CINAHL, pre-print servers (e.g., medRxiv), Google Scholar; 1^st^ March 2020 - 30^th^ November 2021	N = 51. **HCWs:** n = 51	March 2020—May 2021	USA, China, France, Saudi Arabia, Greece, Spain, Cyprus, Germany, Vietnam, Egypt, India, Kuwait, Ghana, Canada, Hong Kong, Palestine, Turkey, Poland, Israel, Congo, Switzerland, South Africa, Japan, Belgium, United Arab Emirates, Germany, Vietnam, Ethiopia, Cyprus, Singapore, Indonesia, Bhutan, Iraq, Qatar, Syria, Jordan, Bahrain, Lebanon	NR
13	Li et al., 2021 [42]	China	Rapid systematic review	PubMed, Embase, Science Direct, Web of Science, China National Knowledge Infrastructure (CNKI), VIP, Wanfang Data; Up to 12^th^ February 2021	N = 13. **HCWs**: n = 13	February 2020—January 2021	USA, China, France, Congo, Turkey, Multicentre: France, Belgium and Canada	JBI for cross-sectional studies NR
14	Lin, Lee et al., 2022 [27]	Malaysia	Systematic review and meta-analysis	Google Scholar, PubMed, Web of Science, Science Direct, Cochrane Library, EBSCO, LILACS, Open Grey; March 2020—October 2021	N = 10. **Dental practitioners:** n = 7 **Dental students:** n = 3	2021	Lebanon, Italy, USA, Israel, Greece, Palestine, Kuwait, Pakistan, India, Multicentre: 22 countries	JBI for cross-sectional studies Moderate risk of bias: n = 4; Low risk of bias: n = 6
15	Lin, Tu et al., 2021 [43]	USA	Rapid systematic review	PubMed, EMBASE, PsycINFO, Google; 1 January 2020 - 20^th^ October 2020	N = 126. **HCWs:** n = 4	**HCWs:** February 2020—September 2020	**HCWs:** Israel, Malta, Hong Kong, Indonesia	NR
16	Luo et al., 2021 [28]	China	Systematic review and meta-analysis	PubMed, EMBASE, Web of Science, Cochrane Library, Chinese National Knowledge Infrastructure (CNKI), Chongqing VIP Chinese Science (VIP), Wanfang Database, China Biomedical Literature Database (CBM); Up to 19^th^ February 2021	N = 9. **HCWs:** n = 9	February 2020—December 2020	USA, China, France, Greece, Multicentre: France, Belgium and Canada	Downs and Black assessment checklist High quality (total score of 5–6): n = 1; Moderate quality (total score of 3–4): n = 8
17	Machado et al., 2021 [49]	The Netherlands	Scoping review	PubMed, Web of Science; 1 January 2020 - 1^st^ August 2021	N = NR. **HCWs:** n = 2	March 2020—October 2020	NR	NR
18	Ngangue et al., 2022 [20]	Burkina Faso	Systematic review	MedLine, CINAHL, EMBASE, Global Health databases; Up to 30^th^ June 2021	N = 23. **HCWs:** n = 9 **Medical students:** n = 2	NR	**All groups:** Africa	Mixed Methods Appraisal Tool for methodological quality of mixed systematic reviews Good quality: n = 20; Moderate quality: n = 2; Poor quality: n = 1
19	Olu-Abiodun et al., 2022 [44]	Nigeria	Rapid systematic review	PubMed, Web of Science, Cochrane Library, EMBASE, African Journals Online (AJOL), Google Scholar, HINARI; January 2020—November 2021	N = 10. **HCWs:** n = 3	**HCWs:** October 2020—March 2021	**HCWs:** Ondo Edo Delta, Abia, Across six geopolitical zones	NR
20	Patwary et al., 2022 [29]	Bangladesh	Rapid Systematic review and Meta-analysis	MedLine (via PubMed), Web of Science, Scopus, Google Scholar; January 2020—August 2021	N = 36. **HCWs**: n = 7 **HC students**: n = 3	**HCWs:** September 2020—April 2021 **HC students:** January 2021—March 2021	**HCWs:** Bangladesh, Egypt, Uganda, India, Nepal **HC students:** Egypt, Uganda, India	JBI (not specified) High quality (≥50%): n = 36
21	Pekcan et al., 2021 [30]	USA	Systematic review and meta-analysis	PubMed; Up to 31^st^ March 2021	N = 49. **HCWs:** n = 12	**HCWs:** March 2020—March 2021	**HCWs:** Israeli, China, France, USA, Italy, Turkey, Multicentre: France, Belgium and Canada; China, India, Indonesia, Singapore, Vietnam and Bhutan	JBI for cross-sectional studies Score of 4: n = 3; Score of 5: n = 7; Score of 6: n = 11; Score of 7: n = 13; Score of 8: n = 15
22	Salomoni et al., 2021 [35]	Italy	Systematic review	PubMed; November 2019—March 2021	N = 100. **HCWs:** n = 21 **University students:** n = 7 **Mixed population (HCWs and Medical students:** n = 1	**HCWs:** February 2020—January 2021 **University students:** June 2020—January 2021 **Mixed population:** August 2020 –September 2020	**HCWs:** USA, Mexico, France, Israel, Italy, Greece, Democratic Republic of Congo, Hong Kong, Nepal, Multicentre: China, India, Indonesia, Singapore, Vietnam and Bhutan; France, Belgium and Canada **University students:** USA, Italy, Poland, Egypt, China **Mixed population:** USA	NR
23	Shakeel et al., 2022 [36]	USA	Systematic review	PubMed, Web of Science, IEEE Xplore, ScienceDirect; 1^st^ January 2020 - 31^st^ July 2021	N = 81. **HCWs:** n = 14 **College students:** n = 3 **Multiple groups (general population, HCWs and HC students:** n = 2 **Dentists, dental surgeons and dental students:** n = 2	**All groups:** 2020–2021	**HCWs:** China, Saudi Arabia, Kuwait, Hong Kong, Italy, Turkey, Cyprus, Greece, France, South Africa, Democratic Republic of Congo **College students:** China, Italy, Egypt **Multiple groups:** Iraq, Israel **Dentists, dental surgeons and dental students:** Israel, USA	NR
24	Snehota et al., 2021 [37]	Czech Republic	Systematic review	PubMed, Web of Science, Scopus; 2019–2020	N = 62. **HCWs:** n = 11 **Students:** n = 5 **Mixed population:** n = 1	**HCWs:** February 2020—September 2020 **Students:** February 2020—September 2020 **Mixed population:** May 2020—August 2020	**HCWs:** Democratic Republic of Congo, France, Germany, Greece, China, Israel, Malta, Nepal, Turkey **Students:** China, Italy, Jordan, Malta, USA **Mixed population:** Germany	NR
25	Troiano & Nardi, 2021 [38]	Italy	Systematic review	PubMed (MEDLINE); Up to November 2020	N = 15. **HCWs:** n = 3 **Students:** n = 1	**HCWs:** February 2020—September 2020 **Students:** NR	**HCWs:** Israel, Malta, Hong Kong **Students:** Italy	NR
26	Ulbrichtova et al., 2022 [31]	Slovakia	Systematic review and meta-analysis	PubMed, Web of Science, Scopus; January 2020 –December 2021	N = 6. **Medical students:** n = 6	February 2020—August 2021	Saudi Arabia, Kazakhstan, India, USA, Texas, Slovakia, Japan	Downs and Black assessment checklist High quality (total score of 5–6): n = 0; Moderate quality (total score of 3–4): n = 6; Low quality (total score of 1–2): n = 0
27	Wake, 2021 [21]	Ethiopia	Systematic review	PsycINFO, HINARI, Cochrane Library, PubMed, EMBASE, African Journals Online, Web of Science, Scopus, Google Scholar; up to 16^th^ July 2021	N = 48. **HCWs:** n = 19 **Non-HCWs:** n = 29	**HCWs:** July 2020—March 2021 **Non-HCWs:** March 2020—March 2021	**HCWs:** France, Ethiopia, India, Saudi Arabia, Cyprus, Asia-Pacific, Bangladesh, Nepal, Italy, Libya, USA, Egypt, Germany, Poland, Multicentre: France, Belgium and Canada **Non-HCWs:** Germany, Belgium, Ethiopia, Bangladesh, Lebanon, Poland, China, Turkey, France, UK, USA, Slovenia, Italy, Malta, Qatar, Jordan, Oman, Mexico, Egypt	NOS for cross-sectional studies High quality (≥5/10): n = 48
28	Wake, 2021 [39]	Ethiopia	Systematic review	PubMed/MEDLINE, HINARI, EMBASE, Google Scholar, Web of Science, Scopus, African journals, Google; 8^th^ May 2021	N = 45. **HCWs:** n = 12 **University students:** n = 3	**HCWs:** 2020–2021 **University students:** 2020–2021	**HCWs:** China, Congo, USA, Israel, Saudi Arabia, France, Vietnam, Pakistan **University students:** China, Italy, Uganda	NOS for cross-sectional studies All studies scored ≥5 out of 10 points
29	Wang & Liu, 2022 [45]	USA	Rapid systematic review	PubMed; Up to 20^th^ February 2021	N = 106. **HCWs:** n = 14 **College students:** n = 5	**HCWs:** 2021 **College students:** 2020–2021	**HCWs:** Los Angeles, New Mexico, Texas, Missouri and Ohio, New York, Illinois, Pennsylvania **College students:** South Carolina, Michigan, Florida and Utah, Michigan	NR
30	Willems et al., 2021 [22]	The Netherlands	Scoping review	CINAHL, APA PsycArticles and APA PsycInfo (via the EBSCO host), Web of Science, Semantic Scholar, Prospero, Outbreak Science, Cochrane, Scopus; 2020 –up to April 2021	N = 26. **HCWs:** n = 26	February 2020—March 2021	USA, Italy, Israel, Spain, France, Congo, Uganda, Turkey, China, Canada, UK, Egypt, Romania, Saudi Arabia, Poland, Multicentre: Belgium, France and Canada; 33 countries worldwide	NR
31	Yasmin et al., 2021 [40]	Pakistan	Systematic review	MEDLINE (PubMed), Cochrane Library, Google Scholar; Up to 17^th^ July 2021	N = 65. **HCWs:** n = 2 **Students:** n = 6	**HCWs:** December 2020—January 2021 **Students:** August 2020—December 2020	**HCWs:** New York, Illinois **Students:** Kansas, New Jersey, Michigan, Florida and Utah, Rhode Island, Southeast Michigan	NR

*Notes*. NR = Not reported; HCW = Healthcare worker; HC = Healthcare; NOS = Newcastle Ottawa Scale; JBI = Joanna Briggs Institute

### 3.3 Characteristics of participants

The sociodemographic characteristics of the populations included in the reviews are outlined in Table 4. Nine reviews focused on HCWs [5, 22–25, 28, 34, 42, 48], three reviews focused on healthcare students [26, 27, 31] and 19 included a mixed population either containing a subset of HCWs [21, 30, 32, 33, 41, 43, 44, 47, 49] or subsets of both HCWs and healthcare students [20, 29, 35–40, 45, 46]. There was large variability in occupational roles reported for the HCWs, but Physicians, Nurses, Dentists, Pharmacists, Midwives, Physiotherapists and General Practitioners were the most commonly cited occupational roles across the reviews. The most frequently reported trainee courses for healthcare students were dentistry, nursing and medicine. The total sample size (where reported) for HCWs was 639,699 (Range = 1941–76,741) and for healthcare students the total number of participants was 70,711 (Range = 934–19,123). Of the 11 reviews reporting sex distributions, all 11 reported a female predominance (>50%) of participants in their primary studies [23–27, 33–35, 38, 40, 48].

**Table 4:** Sociodemographic characteristics of populations included in the Umbrella Review.

No.	Author, year [reference]	Sample size	Occupational Role	Sex	Age (years)	Ethnicity	Vaccine Acceptance Rate
1	Ackah et al., 2021 [24]	**HCWs:** N = 14,132 (Range = 182–2133)	**HCWs**: Doctors (n = 13), Nurses (n = 11), Medical laboratory scientists (n = 3), Pharmacists (n = 10), Allied health (n = 2), Midwives (n = 3), Physiotherapists (n = 2), Dentists (n = 2), Healthcare students (n = 4), Anaesthetists (n = 1), Paramedics (n = 1), Pharmacy students (n = 1), Laboratory Technicians (n = 5), Physicians (n = 1), Health officers (n = 1), Nurses pharmacy (n = 1), Public health (n = 2), Support staff (n = 1) Others (n = 7), All health professionals (n = 2), NR (n = 1)	**HCWs:** Female majority: n = 11; Male majority: n = 9; NR: n = 1	**HCWs:** Pooled Mean age = 33.72 (Pooled Range = 17–55)	**HCWs:** NR	**HCWs:** 48% [95% CI: 38%-57%] **Healthcare students:** 34% [95% CI: 29%-39%]
2	Al-Amer et al., 2021 [32]	**HCWs:** N = 13,059 (Range = 123–3159)	**HCWs:** Physiotherapists (n = 1), Physicians (n = 1), Pharmacists (n = 1), Nurses (n = 3), Assistant nurses (n = 1), Midwives (n = 1), Other HCWs (n = 1), GP or GP trainees (n = 1), Doctors (n = 1)	**HCWs:** NR	**HCWs:** NR	**HCWs:** NR	**HCWs:** Range = 28%-96% **Nurses:** 28% - 65%
3	AlShurman et al., 2021 [46]	**HCWs:** NR **University students and academics:** NR	**HCWs:** Nurses (n = 2), General practitioners and trainees (n = 1), NR (n = 5) **University students and academics:** Healthcare and non-healthcare university students (n = 2), University students, academics and administrators (n = 1), Undergraduate students (n = 1)	**HCWs:** NR **University students and academics:** NR	**HCWs:** NR **University students and academics:** NR	**HCWs:** NR **University students and academics:** NR	**HCWs:** 55.18% (Range = 27.7%-76.9%) **Nurses:** 40% **University students and academics:** 65.78% (Range = 31%-86.1%)
4	Biswas et al., 2021 [5]	**HCWs:** N = 76,741 (Range = 123–16,158)	**HCWs:** NR	**HCWs:** NR	**HCWs:** NR	**HCWs:** NR	**HCWs:** 77.5%
5	Caiazzo & Stimpfel, 2022 [23]	**HCWs:** N = 62,728 (Range = 81–16,292)	**HCWs:** Residents and fellows (n = 1), Physicians (n = 3), Nurses (n = 4), Hospital workers (n = 4), community-based workers (n = 1), Other clinical staff (n = 1), Prescribing clinicians (n = 1), APPs (n = 1), HC workers (n = 5), Emergency department and EMS workers (n = 1), Community-based HC workers (n = 1)	**HCWs:** Combined Female prevalence: 75% (n = 35,084); Combined Male prevalence: 21% (n = 9716)	**HCWs:** <40 years (n = 16,883, 46%; >40 years (n = 14,695, 40%); <45 years (n = 2571,7%); >45 years (n = 1487, 4.1%); NR (n = 1057, 2.9%)	**HCWs:** (where reported) White: n = 30,114 (65.1%); Black: n = 3947(8.5%) Hispanic/Latinx: n = 758 (1.6%); Asian: n = 2316 (5%); Other race: n = 3452 (7.5%)	**HCWs:** 68.8%
6	Dadras et al., 2022 [33]	**HCWs:** N = 21,772 (Range = 187–15,124)	**HCWs:** Physician (n = 2), Nurse (n = 6), Others (n = 3), Doctors (n = 1), University students (n = 1), Rheumatology staff members (n = 1), Physical medicine (n = 1), Dentists (n = 1), Pharmacists (n = 1), NR (n = 1)	**HCWs:** Female majority: n = 8; NR: n = 1	**HCWs:** (where reported: n = 3) Pooled Mean age = 60.73 (Mean Range = 20.24–37.28)	**HCWs:** NR	**HCWs:** Range = 24.4%–82.2%
7	Fattah et al., 2022 [41]	**HCWs:** (where reported) N = 1941	**HCWs:** HCWs (roles not defined) and general population (n = 1), Clinical and non-clinical staff, researchers and trainees (n = 1)	**HCWs:** NR	**HCWs:** NR	**HCWs:** NR	**Scientists & Physicians:** 80.4% **Nurses:** 33.6% **Allied health professionals:** 31.6% **Clinicians:** 32%
8	Galanis et al., 2021 [25]	**HCWs:** N = 50,940 (Range = 208–12,034)	**HCWs:** Physicians (n = 15), Nurses (n = 16), Assistant nurses (n = 4), Paramedical staff (n = 5), Pharmacists (n = 5), Other (n = 14) (Laboratories staff (n = 1), Midwives (n = 5), Physiotherapists (n = 5), administrative staff (n = 5) laboratories staff (n = 5), research staff (n = 5), nurses with midwives (n = 1), technicians and pharmacists (n = 1), technical and administrative staff (n = 1), dentists (n = 1), nurse practitioners (n = 1), assistants (n = 1), certified registered nurse anaesthetists (n = 1), personnel with or without patient contact (n = 3) nurses and others (n = 1))	**HCWs:** Female majority: n = 19; Male majority: n = 4	**HCWs:** NR	**HCWs:** NR	**HCWs:** 63.5%
9	Geng et al., 2022 [26]	**Medical students:** N = 19,123 (Range = 116–6639) **Non-medical students:** N = 21,449 (Range = 99–3226) **Medical and non-medical students:** N = 1971	**Medical students:** Dental students (n = 2), Nursing students (n = 3), Medical and nursing students (n = 1), NR (n = 9) **Non-medical students:** NR (n = 18) **Medical and non-medical students:** NR (n = 1)	**Medical students:** Female majority: n = 11; Male majority: n = 2; NR: n = 2 **Non-medical students:** Female majority: n = 14; Male majority: n = 2; NR: n = 2 **Medical and non-medical students:** Female majority: n = 1	**Medical students:** (where reported: n = 5) Pooled Mean age = 22.74 **Non-medical students:** (where reported: n = 12) Pooled Mean age = 22.39 **Medical and non-medical students:** Pooled Mean age = 20	**Medical students:** NR **Non-medical students:** NR **Medical and non-medical students:** NR	**Medical students:** .74 (95% CI: .67–.81, heterogeneity I2 = 97.1%, P < .001). **Nursing students:** .60 (95% CI: .35–.85, heterogeneity I2 = 99.0%, P< .001) **Dental students:** .60 (95% CI: .54–.67, heterogeneity I2 = 45.7%, P = .159 **Non-medical students:** .72 (95% CI: .66–.78)
10	Hajure et al., 2021 [34]	**HCWs:** N = 33,924 (Range = 140–5287)	**HCWs:** Physicians (n = 8), Nurses (n = 4), Pharmacists (n = 5), Assistant Nurses (n = 1), Midwives (n = 1), Physiotherapists (n = 2), Doctors (n = 5), Nurses and other healthcare workers (n = 1), Dentists (n = 3), General practitioners (n = 1), GP trainees (n = 1), Specialised medical doctors (n = 1), Medical residents (n = 2), Medical doctors (n = 1), Non-MD health professional nurses (n = 1), HC assistants (n = 2), Non-medical students and non-HC workers (n = 1), Frontline HC workers (n = 1), Others (laboratory staff, administrative staff, research staff) (n = 1), Others (n = 3), Scientists and physicians (n = 1), Administration and management (n = 1), Ancillary services (n = 1), Technical support (n = 1), Allied health professionals (n = 1), Master’s-level clinicians (n = 1), Public safety and spiritual care (n = 1), Direct patient care providers (DPCPs) (n = 1), Direct medical providers (n = 1), Others without direct patient contact (n = 1), Graduate sanitary (n = 1), Nurses and midwives (n = 2), HC diagnostic staff (n = 1), Medical students (n = 2), HCWs (n = 1), Junior doctors (n = 1), HC students (n = 1), Dental hygienists (n = 1), Specialists (n = 1), HC managers (n = 1), Nurses and orderlies (n = 1), Environmental service workers (n = 1), Other HC workers (n = 1), Allied health workers (n = 1), Nursing staff (n = 2) Paramedics (n = 1), Paramedical staff (n = 1), Nurses or nursing assistant (n = 1), General workers (n = 1), Medical and dental officers, postgraduates (n = 1), NR (n = 2)	**HCWs:** Female majority: n = 17; Male majority: n = 6; NR: n = 1	**HCWs:** NR	**HCWs:** NR	NR
11	Joshi et al., 2021 [47]	**HCWs:** NR	**HCWs:** Doctors and nurses (n = 1), Nurses (n = 1), NR(n = 1)	**HCWs:** NR	**HCWs:** NR	**HCWs:** NR	**Nurses:** Range = 40%-61% **HCWs:** Range = 77%-78%
12	Khubchandani et al., 2022 [48]	**HCWs:** N = 41,098 (Range = 51–9701)	**HCWs:** Nurses (n = 7), Mixed samples of nurses and other HC professionals (roles not specified) (n = 44)	**HCWs:** Female majority: n = 48	**HCWs:** NR	**HCWs:** NR	**Nurses:** 20.7%
13	Li et al., 2021 [42]	**HCWs:** N = 31,933 (Range = 168–8243)	**HCWs:** Nurses (n = 7), Medical students (n = 3), Physicians (n = 3), Pharmacists (n = 1), Assistant nurses (n = 1), Midwives (n = 3), Physiotherapists (n = 1), other HCWs (n = 2), Doctors (n = 1), Laboratory technicians (n = 1), General Practitioners (n = 1), Clinical and non-clinical staff (n = 1), Researchers and Trainees (n = 1), Nursing home staff (n = 1), Resident (n = 1), Student Nurses (n = 1), Full-time faculty and clinical adjunct faculty (n = 1), Clinical and non-clinical staff (n = 1), Prescribing clinicians (n = 1) Other personnel with direct patient contact (n = 1), personnel without patient contact (n = 1)	**HCWs:** NR	**HCWs:** NR	**HCWs:** NR	**HCWs:** Range = 27.7%-77.3%
14	Lin, Lee et al., 2022 [27]	**Dental practitioners:** N = 12,585 (Range = 250–6639)	**Dental professionals and dental students**: General Dental Practitioners (n = 7), Dental Specialists (n = 3, Dental Students (n = 3), Postgraduate Dental Students (n = 2), Medical Physicians (n = 2), Pharmacists (n = 2), Nurses (n = 1), Lab technicians (n = 1)	**Dental professionals and dental students**: Female majority: n = 6; Male majority: n = 4	**Dental professionals and dental students**: Pooled mean age (where reported: n = 6) = 33.56 (Mean Range = 22.06–44.7)	**Dental professionals and dental students**: NR	**Dental professionals:** 81.1% (Range = 78.8%-86.3%) **Dental students:** 60.5% (Range = 58.0%-61.9%)
15	Lin, Tu et al., 2021 [43]	**HCWs:** N = 4817 (Range = 806–1941)	**HCWs:** Nurses (n = 1), NR (n = 1)	**HCWs:** NR	**HCWs:** NR	**HCWs:** NR	NR
16	Luo et al., 2021 [28]	**HCWs:** N = 24,952 (Range = 461–8243)	**HCWs:** HCWs at university hospital and centre for COVID-19 diagnosis (n = 1), Nurses (n = 2), HCWs in 5 public hospitals (n = 1), HCWs at an academic medical centre (n = 1), HCWs at 5 major hospital systems (n = 1), Nursing home staff (n = 1), General practitioners and nurses (n = 1), Hospital staff and CDC’s staff (n = 1)	**HCWs:** NR	**HCWs:** NR	**HCWs:** NR	**HCWs:** 51%
17	Machado et al., 2021 [49]	**HCWs:** NR	**HCWs:** NR	**HCWs:** NR	**HCWs:** NR	**HCWs:** NR	**Physicians:** 80%
18	Ngangue et al., 2022 [20]	**HCWs:** NR **Medical students:** NR	**HCWs:** NR **Medical students:** NR	**HCWs:** NR **Medical students:** NR	**HCWs:** NR **Medical students:** NR	**HCWs:** NR **Medical students:** NR	NR
19	Olu-Abiodun et al., 2022 [44]	**HCWs:** n = 3256 (Range = 422–1740)	**HCWs:** NR	**HCWs:** NR	**HCWs:** NR	**HCWs:** NR	**HCWs:** Range = 32.5%-55.5%
20	Patwary et al., 2022 [29]	**HCWs:** N = 3154 (Range = 187–831) **Healthcare students:** N = 3801 (Range = 600–2133)	**HCWs:** NR **Healthcare students:** NR	**HCWs:** NR **Healthcare students:** NR	**HCWs:** NR **Healthcare students:** NR	**HCWs:** NR **Healthcare students:** NR	**HCWs:** 41.84% (Range = 21.04%-70.17%) **Healthcare students:** 53.85% (Range = 34.79%-89.42%)
21	Pekcan et al., 2021 [30]	**HCWs:** N = 25,192 (Range = 47–12,034)	**HCWs:** NR	**HCWs:** NR	**HCWs:** NR	**HCWs:** NR	**HCWs:** 70% (95% CI—0.59–0.81)
22	Salomoni et al., 2021 [35]	**HCWs:** N = 61,427 (Range = 230–16,292) **Students:** N = 13,211 (Range = 168–6922) **Mixed population:** N = 1212	**HCWs:** Nurses (n = 1), Dentists (n = 1), Firefighters (n = 1), NR (n = 19) **University students:** Medical students (n = 2, University students (n = 4), NR (n = 1) **Mixed population:** Nurses and Nursing students (n = 1)	**HCWs:** Female majority: n = 14; Male majority: n = 4; NR: n = 3 **University students:** Female majority: n = 6; NR: n = 1 **Mixed population:** Female majority: n = 1	**HCWs:** Range = 21–70 **University students:** Range = 19–23 **Mixed population:** NR	**HCWs:** NR **University students:** NR **Mixed population:** NR	**HCWs:** 67.72% (Range = 27.7–96.2) **University students:** 82.37% (Range = 34.9–98.0) **Mixed population:** 60.0%
23	Shakeel et al., 2022 [36]	**HCWs:** N = 12,601 (Range = 343–2047) **Students**: N = 3198 (Range = 330–2133) **Multiple groups:** N = 2068 (Range = 388–1680) **Dental surgeons and students:** N = 754 (Range = 248–506)	**HCWs:** Nurses (n = 3), Nurses and midwives (n = 1), NR (n = 10) **College students:** College students (n = 1), University students (n = 1), Medical students (n = 1) **Multiple groups:** HCWs, General population and Healthcare students (n = 1), Doctors, General population and Nurses (n = 1) **Dentists, dental surgeons and dental students:** Dentists and dental surgeons (n = 1), Dental students (n = 1)	**HCWs:** NR **College students**: NR **Multiple groups:** NR **Dentists, dental surgeons and dental students:** NR	**HCWs:** NR **College students**: NR **Multiple groups:** NR **Dentists, dental surgeons and dental students:** NR	**HCWs:** NR **College students**: NR **Multiple groups:** NR **Dentists, dental surgeons and dental students:** NR	**HCWs:** 65.1% (Range = 30–91.5%) **College students:** 71% (Range = 36.4–90.5%) **Multiple groups:** 67.0% (Range = 61.1–78.1) **Dentists, dental surgeons and dental students:** 70.5% (Range = 56–85)
24	Snehota et al., 2021 [37]	**HCWs:** N = 10,878 (Range = 230–2827) **Students:** N = 2709 (Range = 167–852) **Mixed population:** N = 1457	**HCWs:** Nurses (n = 2), General practitioners and their trainees (n = 1), NR (n = 8) **Students:** University students (n = 2), University students (medicine and dentistry) (n = 1), University students and staff of faculties of medicine, dentistry and health sciences (n = 1), Medical students (n = 1) **Mixed population:** HCW and university students (n = 1)	**HCWs:** NR **Students:** NR **Mixed population:** NR	**HCWs:** NR **Students:** NR **Mixed population:** NR	**HCWs:** NR **Students:** NR **Mixed population:** NR	**HCWs:** 69.2% **University students:** 76.9% **Mixed population:** 73.0%
25	Troiano & Nardi, 2021 [38]	**HCWs:** N = 3749 (Range = 806–1941) **Students:** N = 934	**HCWs:** Healthcare personnel or general population (n = 1), Nurses (not retired or working in administrative or academic positions) (n = 1), NR (n = 1) **Students:** NR (n = 1)	**HCWs:** Female majority: n = 1; NR: n = 2 **Students:** Female majority: n = 1	**HCWs:** 18–39 years = 59%; >40 years = 41%; NR: n = 2 **Students:** M = 23.6 (SD = 4.9)	**HCWs:** NR **Students:** NR	
26	Ulbrichtova et al., 2022 [31]	**Medical students:** N = 4118 (Range = 204–1228)	**Medical students:** NR (n = 6)	**Medical students:** NR	**Medical students:** NR	**Medical students:** NR	**Medical students:** 61.9% (95% CI, 39.7–80.1%) (Range = 2.0%-91.9%)
27	Wake, 2021 [21]	**HCWs:** N = 21,654 (Rang = 200–5287) **Non-HCWs:** N = 96,530 (Range = 90–32,361)	**HCWs:** Health professionals (n = 1), Medical and dental professionals (n = 1), healthcare providers (n = 1), Nurses and midwives (n = 1), physicians and paramedics (n = 1), Healthcare personnel (n = 2), NR (n = 12) **Non-HCWs:** Hospital employees (n = 1), Hospital staffs (n = 1), Adult population (n = 19), Medical students (n = 1), College students (n = 1), Cancer patients (n = 1), Nonmedical students (n = 1), Breast cancer patients (n = 1), Nursing college students (n = 1), Adolescent population (n = 1), People experiencing homelessness (n = 1)	**HCWs:** NR **Non-HCWs:** NR	**HCWs:** NR **Non-HCWs:** NR	**HCWs:** NR **Non-HCWs:** NR	**HCWs:** Range = 21%-95%
28	Wake, 2021 [39]	**HCWs:** N = 41,176 (Range = 410–12,034) **University students:** N = 8257 (Range = 600–6922)	**HCWs:** Clinical and nonclinical staff (n = 1), Nurses (n = 2), dental residents and oral medicine specialists (n = 1), NR (n = 8) **University students:** University students (n = 2), Medical students (n = 1)	**HCWs:** NR **University students:** NR	**HCWs:** NR **University students:** NR	**HCWs:** NR **University students:** NR	**HCWs:** 61.10% (Range = 36%-85%) **University students:** 67.4% (Range = 37.3%-86.1%)
29	Wang & Liu, 2022 [45]	**HCWs:** N = 27,500 (Range = 240–5287) **College students:** N = 2182 (Range = 167–1062)	**HCWs:** Nursing staff and registered nurses (n = 1), Clinical and non-clinical staff (n = 1), Resident and practicing physicians (n = 1), Licensed pharmacists (n = 1), NR (n = 10) **College students:** Dental students and medical students (n = 1), Medical students (n = 1), NR (n = 3)	**HCWs:** NR **College students:** NR	**HCWs:** NR **College students:** NR	**HCWs:** NR **College students:** NR	**HCWs** Direct Medical providers (e.g. physician and resident): 80.4% Direct patient providers (e.g. nurse, long-term care staff and patient care technician): Range = 25.2%-52.5%
30	Willems et al., 2021 [22]	**HCWs:** N = 43,199 (Range = 24–16,158)	**HCWs:** Specialised Medical Doctor (n = 1), Medical Resident (n = 1), Medical Doctor (n = 2), General Practitioner (n = 1), GP Trainee (n = 1), Non-MD health professional (n = 1), Doctors (n = 2), Nurses (n = 4), Medicine (n = 1), Nursing (n = 1), Other HCWs (n = 3), Physicians (n = 6), Pharmacists (n = 2), Assistant Nurses (n = 1), Midwives (n = 1), Physiotherapists (n = 1), Nurses and other HCWs (n = 1), Nurse/Midwife (n = 1), Student: Medicine and Nurse (n = 1), Paramedic/EMT (n = 1), Other practitioner (n = 1), PA/NP (n = 1), Other health professionals (n = 1), Nursing (n = 1), Medical students (n = 2), Direct patient care providers (n = 1), Direct medical providers (n = 1), Administrative staff (n = 1), Others without direct patient contact (n = 1), HC assistants (n = 1), Nurses & medical/non-medical personnel (n = 1) HCWs for people with intellectual disabilities (n = 2), NR (n = 9)	**HCWs:** NR	**HCWs:** NR	**HCWs:** NR	**HCWs:** Range = 27.7%-82.95%
31	Yasmin et al., 2021 [40]	**HCWs:** N = 4876 (Range = 428–4448) **Students:** N = 4711 (Range = 167–3292)	**HCWs:** Children’s hospital staff (n = 2), NR (n = 1) **Students:** Employees or students at a medical centre (n = 1), Dental students (n = 2), Students at Health Services and College of Pharmacy attending influenza clinics (n = 1), Medical students (n = 1), NR (n = 1)	**HCWs:** Female majority: n = 2 **Students:** Female majority: n = 5; NR: n = 1	**HCWs:** NR **Students:** NR	**HCWs:** (where reported) White = 24% **Students:** (where reported) White = 69.2%	**HCWs:** 45%-90.10%

*Note*. NR = Not reported; HCW = Healthcare worker; HC = Healthcare.

### 3.4 Critical appraisal of included studies

Results from the JBI quality assessment showed that 15 reviews were of ’*strong quality*’ (score: ≥10), seven were of ’*moderate quality*’ (score: 5–9), five were of ’*low quality*’ (score: 0–4) and four were of ’*very low quality*’ (score: ≤0) (S1 Table). Common quality problems were related to a lack of critical appraisal tools and assessment rigour, lack of strategies to minimise errors during data extraction and no assessment of publication bias. The inter-rater agreement score, assessed using Cohen’s Kappa coefficient, was considered ’*moderate*’ (***κ*** = 0.45; 95% CI 0.31–0.60) [16].

### 3.5 Assessment of overlap

The degree to which the 31 reviews included in this Umbrella Review contained the same primary studies was assessed using the CCA method [19]. A total of 462 primary studies (N) were cited across the 31 reviews (C). Of these, 212 were unique citations (R). Results from the CCA calculation ((N–R)/((RxC)–R)) found there was a slight overlap between the reviews (CCA = 3.93%) (S2 Data).

### 3.6 Findings of the review

#### 3.6.1 Vaccine acceptance rates

Vaccine acceptance rates (as a pooled average) were reported in 19 studies [5, 23–31, 35–37, 39, 41, 45, 46, 48, 49] (Table 4). For HCWs, acceptance rates ranged from 20.7% [48] to 81.1% [27]. When broken down by occupational role, Nurses (Pooled average range = 20.7%-40%) and Allied health professionals (Pooled average = 31.6%) had the lowest acceptance rates. In contrast, Dental practitioners (Pooled average = 81.1%), Direct medical providers (Pooled average = 80.4%) and Physicians (Pooled average = 80%) had the highest acceptance rates. For healthcare students, acceptance rates ranged from 34% [24] to 82.4% [35]. Nursing and Dental students both had an acceptance rate of 60% [26].

#### 3.6.2 Determinants of vaccine hesitancy for HCWs

The factors associated with vaccine hesitancy and vaccine acceptance for HCWs are displayed in Table 5.

**Table 5:** Factors associated with vaccine hesitancy and vaccine acceptance for HCWs.

	Vaccine hesitancy [References]	Vaccine acceptance [References]
**VACCINE-RELATED FACTORS**		
Side-effects	Concerns, worries and fear about the adverse, long-term side effects of the vaccine [5, 20, 22, 24, 32–34, 36, 40, 42, 46–49]	Less concerns about serious vaccine side effects [36]
	Concerns over side effects on fertility [48]	
	Uncertainty regarding the tolerability of side effects of the vaccine [20]	
Safety	Concerns about the safety of COVID-19 vaccines [5, 22–24, 30, 32–34, 36, 38, 42–48]	Perceived safety of the vaccine [20, 34, 39, 48, 49]
	Lack of vaccine safety data [48]	
Efficacy/Effectiveness	Concerns about the effectiveness or efficacy of the vaccine [5, 22, 24, 36, 43–46, 48]	Perceived efficacy of the vaccine [20, 21, 32–34, 36, 46, 48]
	Doubted or questioned the efficacy, or effectiveness of the vaccine [20, 30, 32, 33, 38, 42, 47]	
	Concerns over the duration and level of protection against infection from the vaccine [20, 33, 48]	
	Concerns over the effectiveness of the vaccine on novel variants [35, 48]	
Quality	Concerns about the quality of the vaccine [33, 47, 48]	
Vaccine development and approval	Rapid development of the vaccine [5, 22, 33, 41, 42, 48, 49]	Confidence in the development process [48]
	Short duration of the clinical trials [20, 24, 33, 34, 48]	Vaccine’s manufacturing country [33]
	Concerns about the rapid approval of the vaccine [42, 48]	
	Distrust of the expedited vaccine production, healthcare policies, and regulatory procedures [34, 42]	
Politicisation	Politicisation of the vaccines [23, 42]	
	Politicisation of the vaccine development process [5]	
Risk of contracting COVID-19	Perceived risk of contracting COVID-19 infection from the vaccine [24, 30, 33, 48]	
Newness	Perceived risk or concerns because of the newness of the vaccine [21, 34, 42, 48]	
Price	Concerns regarding the financial cost of the vaccine [33]	Lack of payment for vaccines [34]
Logistics	Concerns with storage [36]	Easy logistics to get a vaccine [48]
Perceived benefits	Lack of perceived benefits [36, 48]	Perceived benefits of the vaccine [5, 36, 39, 45, 48]
**INFORMATION & INFORMATION SOURCES**		
Perceptions of information	Insufficient information about the vaccine [22, 24, 33, 36, 42, 48]	
	Insufficient information about the side effects of the vaccine [24]	
	Inadequate information to make an informed decision about vaccination uptake [45, 48]	
	Need for more information [42]	
	Concerns over lack of scientific information [48]	
Knowledge and awareness	Lack of knowledge about COVID-19 [48]	Perceived sufficient knowledge about COVID-19 [5, 21, 48]
	Lack of knowledge about the vaccine [33, 48]	Greater knowledge or understanding of COVID-19 vaccines [25, 48]
	Lack of knowledge about the development and Emergency Use Authorisation processes [20, 45]	Interest in vaccine information [29]
	Poor understanding of the need to vaccinate [36]	Understanding the benefits of the vaccine [36]
Misinformation	Misinformation [22, 24, 44, 48]	
	Misinformation on social media [5, 35, 48]	
	Misinformation from media [33, 48]	
	Misinformation about the COVID-19 vaccine [32]	
	Misinformation about vaccine safety and side effects on social networks [36]	
Information sources	Relying on social media [30]	Facebook [21, 34]
	Use of non-authentic information sources [48]	Centres for Disease Control and Prevention website [39]
		Studying scientific literature [36]
		Greek public health authorities [34, 36, 49]
		National or local TV [45]
		National newspaper [45]
		Reliable sources of information [48]
Conflicting information	Unclear information provided by public health authorities [20]	
	Controversies about the existing vaccine side effects [48]	
	Receiving little or conflicting information about vaccines [30]	
**HEALTH FACTORS**		
COVID-19 status	Confirmed or suspected history of COVID-19 [5, 34, 45, 47, 48]	No previous history of contracting COVID-19 [5, 48]
		Previous COVID-19 infection [20, 22, 25, 39, 45, 48]
Health status	Having chronic medical conditions [5, 36, 42, 48]	Having a chronic condition(s) [5, 21, 22, 25, 32, 34, 38, 39, 42, 43, 46–48]
	Having high-risk medical conditions [23, 45]	No chronic disease history [48]
	Perceived to have a poor/fair health status [23]	No comorbidities [48]
Fertility	Being pregnant [33, 45]	
	Trying to conceive [45]	
Vaccination history	Prior hesitancy towards other vaccinations [23, 43]	Previous history of influenza vaccination [5, 21, 25, 28, 30, 32–34, 36, 38, 39, 42, 43, 45–48]
	Not previously vaccinated with an influenza vaccine [22, 29, 34, 36, 45]	Past vaccination behaviours [5, 22, 25, 29, 30, 34, 39, 46, 48]
	Fear of injections [33, 48]	
Alternative medicine	Use of homoeopathy or naturopathy [32]	
	Preference for alternative treatments [22]	
Smoking		Smoking status [45, 48]
		Being a non-smoker [21]
Allergens		Having an allergy [21, 34, 48]
		No allergies [48]
Weight status		Obesity (BMI >30) [45]
Depression		Depression [26, 34]
**SOCIODEMOGRAPHIC FACTORS**		
Gender	Being female [22, 23, 34, 36, 40, 43, 45]	Gender [21, 45]
		Sex [21]
		Being male [5, 22, 23, 28–34, 36, 38–40, 42, 43, 46, 47]
		Being female [20, 23, 29, 34, 36, 48]
		Non-binary female [23]
Ethnicity	Black and/or Hispanic ethnicity [23, 42]	Ethnicity [22, 45]
	Asian ethnicity [23, 45]	Race [21, 45]
	Asian and Latino [45]	White/Asian race [5, 24, 34, 39]
	Hispanic/Latinx [45]	White race [5, 25, 48]
	Black ethnicity [30, 34, 45]	White ethnicity [23, 45]
	Latinx ethnicity [34]	Non-Hispanic White [40]
	Non-Hispanic Black [45]	Asian ethnicity [23]
	Non-Hispanic Asian [45]	Not Hispanic/ Latinx [42]
	Black/African American [40]	Not Black [42]
	Racial minorities [48]	Non-Black race [5]
		European race [5]
Educational attainment	Lower educational attainment [23, 32, 36, 45, 48]	Higher education level [22, 25, 30, 34, 36, 39, 42, 45, 48]
		Having a doctorate or postgraduate education [5]
		Pharmacists with post-doctoral training [45]
Age	Younger age [20, 22, 25, 36]	Age [21, 22, 33, 45]
	Older age [36, 45]	Older age [5, 22, 23, 25, 30, 32, 34, 36, 39, 42, 43, 46–48]
		Increasing age [32, 42]
		Over 60 [22, 30, 42]
		Over 50 [34, 42, 45]
		45 years and older [33, 34]
		Middle aged [21, 48]
		40 and under [22]
		31–40 years and 41–50 years [20]
		Aged 30–39 [43]
		30 years or older [28]
		18–34 age group [29]
		Younger age (<30 years) [34]
		Younger age [5, 21–23, 36, 39, 42, 46, 48]
Income	Lower-income [20, 48]	Annual income [22, 29, 45]
	Annual household income of >$150,000 [45]	Increased income/Higher income level [25, 34, 39, 42]
		Middle or higher income [5]
Geographical location	Upper-middle and lower-middle countries [27]	Residing in high-income countries [27]
	Residing in the Democratic Republic of Congo [35]	Geographical location of residence [21, 22, 45]
	USA [35]	USA [42]
	Southeast Asia [27]	Eastern Asian countries (China, India, Republic of Indonesia, Singapore, Vietnam and Bhutan) [35]
	Europe [27]	Middle East [27]
	South Africa [36]	
Residential setting	Living in rural areas [23]	Rural living [5]
	Crowded places [48]	
	Junior doctors living with their nuclear families [34]	
Marital status	Single [20]	Married [20, 48]
Family status	Having a child [47]	Having a child or children [36, 48]
Personal preferences	Religious/spiritual reasons [22, 42, 48]	No religious beliefs [48]
	Political reasons [48]	Political views [22, 45]
	Personal reasons [48]	Having liberal political views [32, 48]
		Conservative ideology [5]
		Democrat/liberal [42]
**OCCUPATIONAL FACTORS**		
Occupational role	Nurse [20, 22, 23, 25, 29, 33, 36, 41, 45, 46]	Being in the healthcare profession [21, 22, 32–34, 36, 39, 43, 45, 46, 48]
	Assistant nurse [46]	Doctors [20, 22, 32, 34, 36, 41–43, 46]
	Doctor [20, 34]	Physicians [22, 23, 25, 34, 41, 42, 46]
	Allied health professionals [20, 41, 45]	General practitioners [46]
	Paramedical staff [25]	Prescribing clinicians [23, 42, 45]
	Non-clinical role [45]	Pharmacists [45]
	Trainee [45]	ED/ER workers [48]
		Frontline workers [5]
		Clinical workers [5]
		Dentists [25, 29]
		Dental practitioners [27]
		Rehabilitation centre workers [34]
		Non-MD health professionals [21, 34]
		Students [42]
		Graduate students [45]
		Healthcare students [33]
		Working in a medical faculty [42]
		Research scientists [42]
		Scientists [41]
		Working in public/government institutes [29]
COVID-19 duties	Not caring for COVID-19 patients [32, 41]	Involved in the care of COVID-19 patients [29, 32, 39, 41, 42, 48]
		Occupational COVID-19 exposure [34, 42]
		Encountering with suspected or confirmed COVID-19 patients [5, 25, 32, 39, 42, 46, 47]
		Working in a designated COVID-19 hospital [34]
		Whether their colleagues acquired COVID-19 [42]
		Working in an isolated room [46]
		Working in isolation or ICU [5]
Compulsory vaccination	Believe that HCWs must have the freedom of choice to accept or reject the vaccine [33]	Adhering to the compulsory vaccination [22, 36]
	Requested by employers [36]	Recommended vaccines for health professionals [21, 32]
		Vaccination is part of the job [22]
Occupational setting	Working in rural areas [34]	Working in the private sector [32, 39, 42, 46–48]
	Working in the private sector [36]	Working in a private hospital [21]
		Working in the public health sector [42, 48]
		Working in healthcare facilities in urban areas [25]
		Health care facility or clinical work setting [45, 48]
Employment status	Hourly paid employees [45]	Lost job due to COVID-19/unemployment [36, 39, 41, 43]
	Decreased unemployment rate [36]	Paid sick days at job [48]
	Being a retired HCW [32, 43]	
Occupational pandemic management	Satisfied with PPE [48]	Lack of PPE [46]
		Unfavourable attitudes towards workplace infection control policies [46]
		Work stress is associated with unfavourable attitudes towards workplace infection control policies (acts as a mediator) [30]
		Greater stress at work [42, 46]
Work experience		Years of experience [29]
		Less years of work experience [21, 25, 48]
		Higher work experience [48]
Occupational duties		HCWs caring for patients with comorbidities [34]
		HCWs providing direct patient care [42]
		Pharmacists in indirect patient care settings [45]
**TRUST FACTORS**		
Trust in social actors	Lack of trust in the government [23, 24, 30, 36, 42, 48]	Higher levels of trust or confidence in the government [5, 34, 36, 48]
	Lack of trust in the vaccine manufacturer [23, 24, 33, 48]	Higher levels of trust or confidence in vaccine producers [29, 48]
	Lack of trust in the pharmaceutical companies [5, 20, 48]	Higher levels of trust or confidence in pharmaceutical companies [21, 29, 48]
	Lack of trust in the health authorities [5, 30, 42, 48]	Higher levels of trust or confidence in healthcare systems/ authorities [29, 36, 48]
	Lack of trust in the health experts [5, 30, 36, 42]	Higher levels of trust or confidence in non-health leaders/others [48]
	Lack of trust in the scientists [48]	Higher levels of trust or confidence in science [25, 48]
		Higher levels of trust or confidence in mass media [32]
		Higher levels of trust or confidence in other public health websites/providers [48]
Trust in pandemic management		Trust in the accuracy of the COVID-19 measures taken by the government [20, 34]
		High levels of trust in health promotion strategies [36]
		Confidence in the management of the epidemic [21]
Trust in information		Higher trust in information [45]
Trust in vaccines	Lack of trust or confidence in vaccines [48]	Trust and confidence in other vaccines [5, 25, 46, 48]
	Lack of trust or confidence in vaccine safety [36]	
	Distrust foreign vaccine [30, 48]	
General trust	Distrust because minority/ethnicity [22]	
	General lack of trust [22, 24, 42, 44, 48]	
**BELIEF FACTORS**		
Perceptions of risk	Perceived low risk/severity of COVID-19 infection [20, 23, 43, 48]	Higher perceived risk of susceptibility and severity of COVID-19 infection, now and in the future [5, 20, 25, 30, 32–34, 36, 39, 41–43, 45–48]
	Not concerned or afraid to contract COVID-19 [36, 42, 45]	
Vaccination beliefs	Believing COVID-19 vaccine is unnecessary [32, 38, 42, 43, 46–48]	Positive attitude towards a vaccine/COVID-19 vaccine [5, 25, 36, 39, 42, 48]
	Anti-vaccination beliefs [22, 46, 48]	Positive perceptions of vaccine safety [20, 29]
	Preference for natural immunity [29, 33, 42, 48]	Positive perceptions of vaccine efficacy [20, 48]
	Prefer other COVID-19 preventative measures [48]	Beliefs that vaccines offer protection [5, 39, 45]
		Beliefs that a vaccine is needed to end the pandemic [20, 39, 45]
Perceptions of COVID-19	Perceptions that COVID-19 does not exist [5]	Belief that the pandemic is very severe [48]
	Perceptions that COVID-19 symptoms are mild/harmless [20, 30, 33, 48]	Beliefs that COVID-19 existed as a serious disease [5]
		Having a positive attitude towards COVID-19 [32, 56]
Management of pandemic		Beliefs that the vaccine should be compulsory for the public [29, 34]
		Confidence in and expectations about PPE and behaviours [21, 45]
		Belief that isolation and treatment of people infected are effective in reducing the spread of the virus [46]
**EMOTIONAL FACTORS**		
Anxiety	Higher levels of COVID-19-related anxiety [30]	
Doubt	Higher levels of doubt [48]	
Fear	Higher levels of fear [48]	Fear and worry about COVID-19 [5, 22, 25, 26, 32, 34, 36, 39, 42, 46]
	Fear of the unknown [24, 44]	
**SOCIAL FACTORS**		
Concerns regarding transmission		Concerns or fears of transmitting COVID-19 on to family members, relatives, or friends [5, 22, 26, 34, 48]
		Living with elderly relatives, other family members, or individuals at risk of infection [5, 25, 36, 48]
		Being in close contact with a high-risk group [21]
Altruism		Willingness/ desire to protect others (family, friends, community) [45, 48]
		High altruism [32, 48]
		Collective responsibility [5, 22, 25, 39, 46, 48]
Negative exposure	Heard of anyone with a bad reaction to COVID-19 vaccination [20]	Having a family member/friend diagnosed with COVID-19 [20, 22, 48]
		Having a friend or family member who died due to COVID-19 [20, 48]
		Knows someone who died of COVID-19 [45]
Social encouragement	Lack of social pressure [48]	Encouragement from family or friends [5, 33, 36, 48]
		Encouragement from colleagues or supervisors [33, 36]
		Encouragement from experts [36]
		Encouragement from news media [36]
Vaccination recommendations		Vaccination recommendations [21]
		Vaccination recommendations from doctors [48]
		Vaccination recommendations from others [48]
		Receiving vaccination advice from hospitals [29]
**BEHAVIOURAL FACTORS**		
Proactive behaviours		Preventative COVID-19 infection behaviours [34]
		Willingness/ experience of testing for COVID-19 [34, 39]
		Dispensing the vaccine to their children [34]
Recommending vaccination		Recommending the vaccine to parents [34]
		Recommending the vaccine to people over the age of 70 [34]
		Recommending the vaccine to others [5, 29]
Motivations for vaccination		Contribute to herd immunity [45]
		Inspire confidence that the vaccines are safe [45]
**ENVIRONMENTAL FACTORS**		
Environmental situation		Local area epidemic situation [48]
		Living in an area of high mortality from COVID-19 infections [48]
**BARRIER FACTORS**		
Time	No time to take the vaccine [32, 42, 43, 47, 48]	Delay [35]
	Insufficient time for decision-making [34]	
	Prefer to wait until others have received the vaccine first [33, 34]	
Logistics	Logistics to get vaccinated [22]	
	Access [43]	
Other	Perceived barriers [39]	

*Vaccine-related factors*: HCWs who had concerns regarding the adverse long-term side effects of the vaccine [5, 20, 22, 24, 32–34, 36, 40, 42, 46–49], as well as concerns about the tolerability of vaccine side effects [20] and its impacts on fertility [48] were more hesitant to accept a COVID-19 vaccine. As were those who had concerns about the safety [5, 22–24, 30, 32–34, 36, 38, 42–48], quality [33, 47, 48] and effectiveness or efficacy [5, 20, 22, 24, 30, 32, 33, 36, 38, 42–48] of COVID-19 vaccines. Alternatively, HCWs who displayed less concern for serious vaccine side-effects [36] and perceived the vaccine to be safe [20, 34, 39, 48, 49] and effective [20, 21, 32–34, 36, 46, 48] were more accepting of a vaccine. Greater hesitancy was also shown by individuals who had concerns about the rapid development [5, 22, 33, 41, 42, 48, 49] and approval [42, 48] of COVID-19 vaccines, as well as the short duration of clinical trials [20, 24, 33, 34, 48]. Moreover, distrust of the expedited vaccine production, healthcare policies and regulatory policies [34, 42] and perceived politicisation of the vaccines [5, 23, 42] were associated with greater hesitancy in HCWs. Individuals who had confidence in the vaccine development process [48] and trusted the vaccine manufacturing country [33] were more likely to accept a COVID-19 inoculation. Hesitancy was also associated with concerns regarding the financial cost of the vaccine [33] and concerns regarding vaccine storage [36]. A lack of payment [34] and easy logistics to get vaccines [34] was associated with greater vaccine uptake. HCWs who perceived more vaccine benefits [5, 36, 39, 45, 48] were more accepting than those who perceived a lack of benefits [36, 48]. Individuals who perceived greater risks, such as contracting COVID-19 infection from the vaccine [24, 30, 33, 48], were more hesitant to accept an inoculation.

*Information and sources*: HCWs who perceived a lack of information about the vaccine [22, 24, 33, 36, 42, 48] and its side effects [24] exhibited more hesitancy towards a COVID-19 vaccine. As did those concerned about a lack of scientific information [48] or perceived a lack of adequate information available to support an informed decision [48, 45]. HCWs with a lack of knowledge about COVID-19 [48] and COVID-19 vaccines [33, 48] were more hesitant than HCWs who had sufficient knowledge about COVID-19 [5, 21, 48] and COVID-19 vaccines [25, 48]. HCWs who utilised social media [30] or non-authentic information sources [48] were more hesitant than HCWs who retrieved their COVID-19 related information from scientific literature [36], health authorities and associated websites [34, 36, 39, 49], national or local TV [45], national newspapers [45], Facebook [21, 34] or other reliable sources of information [48]. Exposure to misinformation about COVID-19 and the vaccines on social media was associated with vaccine hesitancy [5, 22, 24, 32, 33, 36, 44, 48]. Similarly, receiving conflicting or unclear information about COVID-19 [30] and its side effects [48], especially by public health authorities [20], was also found to contribute to vaccine hesitancy.

*Health factors*: HCWs who had not previously contracted a COVID-19 infection were more accepting of a COVID-19 vaccine [5, 48]. However, a history of COVID-19 infection was associated with both vaccine hesitancy [5, 34, 45, 47, 48] and acceptance [20, 22, 25, 39, 45, 48]. Having a chronic health condition was both a determinant of vaccine hesitancy [5, 23, 36, 42, 45, 48] and acceptance [5, 21, 22, 25, 32, 34, 38, 39, 42, 43, 46–48]. Individuals with no chronic health conditions [48] were more likely to accept a COVID-19 vaccination. HCWs who had previously accepted an influenza vaccination [5, 21, 25, 28, 30, 32–34, 36, 38, 39, 42, 43, 45–48] or other inoculations [5, 22, 25, 29, 30, 34, 39, 46, 48] were more likely to accept a COVID-19 vaccine than HCWs who had not accepted an inoculation [23, 43] such as the influenza vaccine [22, 29, 34, 36, 45] previously. Fear of injections was also associated with hesitancy [33, 48]. An individual’s smoking status [21, 45, 48], allergen history [21, 34, 48], BMI status [45] and depression history [26, 34] were all factors associated with vaccine acceptance. Individuals who were pregnant [33, 45] or trying to conceive [45] were hesitant to accept a COVID-19 inoculation.

*Sociodemographic factors*: This review found that gender [21, 45], specifically, male [5, 22, 23, 25, 28–34, 36, 38–40, 42, 43, 46, 47] and non-binary female [23] was associated with vaccination acceptance. Being female was found to be both a barrier [22, 23, 34, 36, 40, 43, 45] and facilitator [20, 23, 29, 34, 36, 48] of vaccine uptake. An individual’s ethnicity/race [20, 21, 45] contributed to vaccination intentions. In particular, White ethnicity/race [5, 23, 25, 45, 48] (including White/Asian race [5, 34, 39, 42] and Non-Hispanic White [40]) was associated with vaccine acceptance, while Black ethnicity/race [30, 34, 45] (including Black and Hispanic [23, 42], Non-Hispanic Black [45] and Black/African American [40] ethnicities) was associated with vaccine hesitancy. Asian ethnicity was found to be both a barrier [23, 45] (including Asian and Latino [45] and Non-Hispanic Asian [45] ethnicities) and a facilitator [23] of vaccine acceptance. Latinx ethnicity [34] (including Hispanic/Latinx [45] ethnicities) was also associated with vaccine hesitancy. Both younger (<40) and older age (>45) were associated with vaccine hesitancy [20, 22, 25, 36, 45] and acceptance [5, 20–23, 25, 28–30, 32–34, 36, 39, 42, 43, 45–48]. Higher educational attainment [5, 22, 25, 30, 34, 36, 39, 42, 45, 48] was associated with vaccine acceptance and lower educational attainment with vaccine hesitancy [23, 32, 36, 45, 48]. Increased or higher income [5, 25, 34, 39, 42] and lower income [20, 48] were associated with vaccine acceptance and hesitancy, respectively. HCWs residing in upper-middle and lower-middle countries [27], or the Democratic Republic of Congo [35], Southeast Asia [27], Europe [27] and South Africa [36] were more likely to be hesitant towards a COVID-19 vaccine than HCWs residing in high-income countries [27], Eastern Asian countries (China, India, Republic of Indonesia, Singapore, Vietnam and Bhutan) [35] and Middle East countries [27]. Residing in the USA was a barrier [35] and facilitator [42] of vaccine acceptance. HCWs who were married [20, 48] were more accepting of a COVID-19 vaccine than those who were single [20]. Having a child or children was a barrier [47] and a facilitator of vaccine acceptance [36, 48]. An individual’s religious or political beliefs could be both a barrier [22, 42, 48] and a facilitator [5, 22, 34, 42, 45, 48] of acceptance of a COVID-19 vaccination.

*Occupational factors*: Vaccine acceptance was associated with working in public/government institutes [29], a medical faculty [42], or in the healthcare profession [21, 22, 32–34, 36, 39, 43, 45, 46, 48]. In particular, Physicians [20, 22, 23, 25, 32, 34, 36, 41–43, 46], General practitioners [46], Pharmacists [45], prescribing Physicians [23, 42, 45], Emergency department/room workers [48], Clinical or frontline workers [5], Dentists [25, 29], Dental practitioners [27], Rehabilitation centre workers [34], non-MD health professionals [21, 34], Scientists [41] and Research scientists [42] displayed more vaccine acceptance. Whereas Nurses [20, 22, 23, 25, 29, 33, 36, 41, 45, 46], assistant nurses [46], Paramedical staff [25], Allied health professionals [20, 41, 45], Trainees [45] and those in non-clinical roles [45] were found to be more hesitant towards a COVID-19 inoculation. HCWs involved in COVID-19 duties (e.g. caring for COVID-19 patients [29, 32, 39, 41, 42, 48], working in a designated hospital [34]) and were exposed to COVID-19 through patient [5, 25, 34, 39, 42, 47] or colleague interactions [42] were more likely to accept a COVID-19 vaccine. Contrastingly, individuals who did not care for COVID-19 patients [32, 41] were more hesitant. HCWs who displayed unfavourable attitudes towards workplace infection control policies [30, 46] and a lack of personal protective equipment (PPE) [46] were more accepting of a vaccine than those HCWs who were satisfied with workplace PPE [48]. Attitudes toward compulsory vaccination for HCWs were both a barrier [33, 36] and facilitator [21, 22, 32, 36] of vaccine uptake, depending upon an individual’s level of agreement. Retired [32, 43] and hourly paid HCWs [45] were less accepting of a vaccine. HCWs unemployed due to COVID-19 were more accepting of a vaccine [36, 39, 41, 43].

*Trust factors*: HCWs that had a lack of trust in key social actors, such as the government [23, 24, 30, 36, 42, 48], vaccine manufacturers [23, 24, 33, 48], pharmaceutical companies [5, 20, 48], health authorities [5, 30, 42, 48], health experts [5, 30, 36, 42] and scientists [48] were more hesitant to accept a COVID-19 vaccine. Whereas HCWs that had higher levels of trust in these social actors, as well as non-health leaders [48], mass media [32], science [25, 48] and public health websites or providers [48], were more accepting of inoculation. Additionally, higher levels of trust in pandemic management [20, 21, 34] and health promotion strategies [36] were associated with vaccine acceptance. Individuals with a lack of trust or confidence in vaccines [48] and their safety [36] displayed more hesitancy than individuals that were more trusting of vaccines [5, 25, 46, 48].

*Belief factors*: Individuals that perceived themselves to be at higher risk of contracting a severe COVID-19 infection [5, 20, 25, 30, 32–34, 36, 39, 41–43, 45–48] were more accepting of a vaccination than those perceiving themselves to be less susceptible [20, 23, 43, 48] or less concerned with contracting COVID-19 [36, 42, 45]. HCWs that had negative perceptions regarding COVID-19, for example, that the disease does not exist [5], that symptoms are mild [20, 30, 33, 48] and a vaccine is not necessary [32, 38, 42, 43, 46–48], were also more hesitant to accept a COVID-19 inoculation. As were those who held anti-vaccination beliefs [22, 46, 48] and preferred natural immunity [29, 33, 42, 48] or other COVID-19 preventative measures [48]. Alternatively, individuals with positive perceptions of COVID-19 [32, 46] and the benefits [5, 20, 39, 45], safety [20, 29] and efficacy [20, 48] of a COVID-19 vaccine [5, 25, 36, 39, 42, 48] were more likely to accept an inoculation. HCWs that perceived COVID-19 to be a serious disease [5] and believed in the importance of pandemic management strategies, such as compulsory vaccination for the public [29, 34], isolation as an effective transmission reduction strategy [46] and had confidence in and expectations about PPE and other preventative behaviours [24, 45] were more accepting of a COVID-19 vaccine.

*Emotional factors*: Higher levels of negative emotional responses (e.g. anxiety, doubts, fear and worry) were found to be a driver of vaccine hesitancy for some individuals [24, 30, 44, 48] and vaccine acceptance for others [5, 22, 25, 26, 32, 34, 36, 39, 42, 46].

*Social factors*: Individuals who expressed high levels of altruism towards protecting others [32, 45, 48] or perceived vaccination as a collective responsibility [5, 22, 25, 39, 46, 48] were more likely to accept an inoculation. HCWs concerned about transmitting COVID-19 to family members, relatives or friends [5, 22, 26, 34, 48], or those who lived with elderly relatives [5, 25, 36, 48] or vulnerable individuals [5, 21, 25, 36, 48] were more accepting of a COVID-19 vaccine. As were those individuals who had a family member or friend who had been diagnosed with [20, 22, 48] or had died from COVID-19 [20, 45, 48]. Vaccination recommendations from healthcare professionals [29, 48] or encouragement from social networks [5, 33, 36, 48], experts [36], or news media [36] all contributed to vaccine acceptance. In contrast, individuals experiencing less social pressure were more likely to be hesitant to accept an inoculation [48]. Similarly, individuals were more likely to be hesitant if they had been exposed to anecdotes regarding negative reactions to a COVID-19 vaccination [20].

*Behavioural factors*: HCWs who exhibited proactive behaviours, such as engaging in preventative behaviours [34] and testing procedures [34, 39], were more accepting of vaccination, as were those who recommended the vaccine to parents [34], elderly individuals [34] and the general population [5, 29] and had dispensed the inoculation to their children [34]. Individuals more accepting of a COVID-19 vaccine were more likely to do so to inspire confidence in vaccine safety [45] and contribute to herd immunity [45].

*Environmental factors*: Vaccine acceptance was associated with the severity of the local epidemic situation and local COVID-19 mortality figures [48]. HCWs who were hesitant to accept the vaccine reported a lack of time to seek out vaccination [32, 42, 43, 47, 48] or make a decision [34] and preferred to wait until others had received the vaccine first [33, 34]. An initial delay in accepting a vaccine was also associated with vaccine acceptance at a subsequent timepoint [35].

#### 3.6.3 Determinants of vaccine hesitancy for healthcare students

The amalgamation of results from the ten reviews that included populations of healthcare students found various factors associated with hesitancy or acceptance of a COVID-19 vaccine (Table 6).

**Table 6:** Factors associated with vaccine hesitancy and vaccine acceptance for healthcare students.

	Vaccine hesitancy [References]	Vaccine acceptance [References]
**VACCINE RELATED FACTORS**		
Side effects	Worries and concerns about the adverse side effects of the vaccine [20, 26, 40, 45, 46]	Less concern for serious vaccine side effects from a COVID-19 vaccine [45]
Safety	Fears of vaccine safety [36, 40]	
Efficacy/Effectiveness	Concerns about the effectiveness or efficacy of the vaccine [20, 40]	Perceived efficacy of the vaccine [39, 46]
Price	Fee-based vaccine [35]	Free vaccine [35]
Perceived benefits		Perceived benefits of the vaccine [36]
**INFORMATION & INFORMATION SOURCES**		
Perceptions of information	Insufficient information about the vaccine [20, 36, 40]	
	Insufficient information/data about the side effects of the vaccine [20, 36]	
Knowledge	Lack of knowledge regarding COVID-19 [36]	Perceived sufficient knowledge about COVID-19 [26, 29, 45]
		Those who realised the importance of COVID-19 [26]
Information sources	Getting information about the COVID-19 vaccine from social media [26]	Use of social media for COVID-19 vaccine-related information [39]
		Scientists as an information source [45]
		Pharmaceutical companies as an information source [45]
**HEALTH FACTORS**		
COVID-19 status	Previously infected with COVID-19 [26]	
Health status	Perceived good health status [46]	Average to very good self-perception of health status [29]
		Immunocompromised person [46]
		Previous illness [40]
Vaccination history	Prior hesitancy towards other vaccinations [26]	Receiving any vaccine in the past five years [29, 39]
		Likelihood of influenza vaccination [46]
Alternative medicine	Use of homoeopathy and naturopathy [46]	
**SOCIODEMOGRAPHIC FACTORS**		
Gender	Female [31]	Male [20, 21, 29, 45, 46]
		Female [39]
Ethnicity	Black/African American [40]	Non-Hispanic White [40]
		Underrepresented minorities [45]
Age		Older age [40, 45, 46]
Income		Higher-income [36]
Socioeconomic status		Higher socioeconomic status [36]
Geographical location	Location of residence (Egypt) [35]	Location of residence (USA) [35]
Marital status		Single [20, 29, 39]
**EDUCATIONAL FACTORS**		
Student status	Dental students [40]	Healthcare and non-healthcare students [46]
	Non-medical students [35]	Medical students [35, 40]
	Program of study [36]	Pharmacy student [29]
		Being an academic, student or support staff [46]
		Being in a medical faculty [46]
Year of Study	First- and second-year students [31]	Third year of study and above [29, 31]
		Graduate student [29, 45]
Residency setting	Place of residence [36]	
**TRUST FACTORS**		
Trust in social actors		Higher levels of trust or confidence in the government [36, 46]
		Higher levels of trust or confidence in the mass media [45, 46]
		Higher levels of trust or confidence in social media [45]
		Higher levels of trust or confidence in the healthcare systems/agencies [45, 46]
		Higher levels of trust or confidence in public health experts [45]
		High levels of trust in health promotion strategies [36]
		Higher levels of trust or confidence in scientists [45]
		Higher levels of trust or confidence in pharmaceutical companies [45]
**BELIEF FACTORS**		
Perceptions of risk		Higher perceived risk of susceptibility and severity of COVID-19 infection, now and in the future [20, 29, 39]
Vaccination beliefs	General opposition to vaccines [46]	Perceived importance of the vaccine [46]
	Disagreement towards the introduction of compulsory vaccination [26, 31]	Agreement toward the introduction of compulsory vaccination [26, 31]
Beliefs in conspiracy theories	Conspiracy beliefs [46]	
Perceptions of barriers	Perceptions of barriers [36]	
**EMOTIONAL FACTORS**		
Negative emotions		Concerns about COVID-19 infection [26]
**SOCIAL FACTORS**		
Concerns regarding transmission		Individuals with family members who have compromised immune systems [46]
Exposure		Presence of confirmed COVID-19 infection in a close social network [29]
Social influences		Family members vaccinated [40]
Social isolation		Suffering from distance to friends during pandemic containment [26]
**ENVIRONMENTAL FACTORS**		
Environmental situation		Changes in the physical environment [45]
		Fear of increasing death rates [26]

*Sociodemographic factors*: This review found that female students [31] were more hesitant than male students [20, 21, 29, 45, 46]. Although, one review reported that being female was a factor associated with acceptance [39]. With regards to ethnicity, Black/African American students [40] displayed more hesitancy towards the vaccine than non-Hispanic White students [40] or those from underrepresented minorities [45]. Additional sociodemographic factors associated with vaccine acceptance in this population were older age [40, 45, 46], having a higher income [36], higher socioeconomic status [36] or whose marital status was single [20, 29, 39]. Vaccine acceptance was lower in students residing in Egypt [35] and higher in students residing in the USA [35].

*Educational factors*: Dental students [40] and non-medical students [35] exhibited more hesitancy, whereas pharmacy students [29], medical students [35, 40] and both healthcare and non-healthcare students [46] were more accepting of a vaccine. Other vaccine-accepting populations were academics, students, support staff [46] and individuals working in a medical faculty [46]. Additionally, students in their first or second year of study [31] were more hesitant than students in their third year (or above) of study [21, 29] or were graduate students [29, 45]. Healthcare students who disagreed with compulsory vaccination for healthcare professionals were more hesitant than those in support of a compulsory COVID-19 vaccine [26, 31].

*Health factors*: Perceptions of good health were found to determine vaccine hesitancy [46] and acceptance [29]. Alternatively, students with a history of previous illnesses [40] who were immunocompromised or had a family member with a compromised immune system [46] were more likely to accept a COVID-19 vaccine. Individuals previously infected with COVID-19 [26] were hesitant, as were those who engaged in homoeopathy or naturopathy practices [46]. Students who exhibited general opposition to vaccines [46] and previous hesitancy towards other vaccinations [26] were also more likely to exhibit hesitancy towards a COVID-19 vaccine. Alternatively, students who had received an inoculation in the past five years [29, 39] or were willing to accept an influenza shot [46] were more likely to accept a COVID-19 inoculation. Individuals who perceived themselves at higher risk of a severe COVID-19 infection now and in the future were more likely to accept a COVID-19 inoculation [20, 29, 39].

*Vaccine-related factors*: Healthcare students that expressed more worries and concerns about the efficacy of the vaccine [20, 40], the safety of the vaccine [36, 40] and the adverse side effects from the vaccine [20, 26, 40, 45, 46] were more hesitant to accept the COVID-19 vaccine. Alternatively, healthcare students less concerned about adverse side effects [45] and who perceived the vaccine to be effective [39, 46] and beneficial [36] were more accepting of an inoculation. Additionally, a fee-based vaccine was associated with more hesitancy than a freely available vaccine [35].

*Information and sources*: Students who perceived a lack of information about the vaccine [20, 36, 40] and data on its side effects [20, 36] exhibited more hesitancy towards a COVID-19 vaccine. Individuals who perceived sufficient knowledge regarding COVID-19 [26, 29, 45] and realised the importance of COVID-19 [26] and an associated vaccine [45] were more likely to accept an inoculation. Evidence suggests that using social media for COVID-19 vaccine-related information was found to be both a barrier [26] and a facilitator [39] of vaccine acceptance. Furthermore, the use of scientists [45] or pharmaceutical companies [45] as a source of information about COVID-19 vaccines was associated with vaccine uptake. Contrastingly, exposure to conspiracy theories was associated with greater hesitancy [46].

*Trust factors*: Higher levels of vaccine acceptance were associated with higher levels of trust or confidence in the government [36, 46], mass media [45, 46], social media [45], healthcare systems and agencies [45, 46], public health experts [45], health promotion strategies [36], scientists [45] or pharmaceutical companies [45].

*Social factors*: Social factors associated with vaccine acceptance were the presence of COVID-19 infection within a close social network [29], having family members that had received a COVID-19 vaccine [40] and suffering from distance to friends during pandemic containment [26]. Additionally, students expressing fear of death rates were more accepting of a vaccine [26].

#### 3.6.4 Results from meta-analyses

Four reviews conducted a meta-analysis exploring the role sociodemographic variables have on determining COVID-19 vaccine hesitancy or acceptance in HCWs or healthcare students. For HCWs, the only variables found to significantly predict vaccine acceptance was being male [28, 29], aged 30 years or older [28], having a history of prior influenza vaccination [28] and perceived risk of COVID-19 infection [29] (S2 Table). Unlike HCWS, age [26] and gender [26, 31] were not significant predictors of vaccine acceptance for healthcare students. Factors related to vaccine uptake for this population were being a student in a medical field [26], supporting compulsory vaccination [26, 31], perceived risk of COVID-19 infection [26], perceived sufficient knowledge of COVID-19 [26] and recognition of the importance of COVID-19 vaccination for individuals [26]. Alternatively, healthcare students previously infected with COVID-19, those worried about the vaccine’s adverse effects, or those with negative attitudes toward compulsory vaccination [26] were significantly more hesitant to accept a COVID-19 vaccination (S3 Table).

## 4. Discussion

This is the first comprehensive Umbrella Review that has compiled the evidence pertaining to vaccine hesitancy and acceptance of a COVID-19 vaccine for HCWs and healthcare students. This review found that vaccine hesitancy rates were variable across occupational roles, whereby some professions, such as physicians [49] and dental practitioners [27], were more accepting of a COVID-19 vaccine than other occupations, for instance, nurses [41, 46, 48]. In several reviews where acceptance rates were compared to the general public, HCWs displayed more hesitancy toward the COVID-19 vaccine [28, 37, 29]. Most reviews exploring hesitancy in healthcare students found that medical students were less hesitant toward a COVID-19 vaccine than non-medical students [22, 35, 46]. Dental students exhibited more hesitancy than dental practitioners [27], and nursing students displayed higher acceptance rates than nurses [26]. The variability in hesitancy rates across various occupational groups suggests a need for tailored intervention strategies that address the barriers contributing to vaccine hesitancy for each occupational group. Previous research has found that educational sessions delivered by less hesitant HCWs effectively increased vaccine acceptance rates in previously hesitant HCWs [50, 51]. Implementing COVID-19-related information sessions across hospitals or departments that enable HCWs, such as nursing staff, to discuss concerns and questions with other HCWs (e.g. physicians) may be efficacious in improving COVID-19 vaccine uptake in more hesitant groups of HCWs. Similarly, educational interventions with healthcare students throughout their training courses may be a way to help address hesitancy and promote and maintain vaccine acceptance throughout their medical careers and beyond.

Many factors associated with vaccine hesitancy were identified, one of the most commonly explored and reported drivers was related to sociodemographic factors. Although there is an abundance of evidence pertaining to gender and age, the evidence is inconsistent. Regarding gender, being a male HCW was associated with vaccine acceptance [5, 22, 23, 25, 28–34, 36, 38–40, 42, 43, 46, 47], and was a significant predictor of COVID-19 vaccine uptake in two meta-analyses [28, 29]. However, being a female HCW was associated with both vaccine hesitancy [22, 23, 34, 36, 40, 43, 45] and vaccine acceptance [20, 23, 29, 34, 36, 48]. This pattern was also identified in healthcare students, whereby males were more accepting of an inoculation [20, 21, 29, 45, 46] and being female was both a barrier [31] and facilitator [39] of vaccination uptake. However, the results from two meta-analyses found that gender was not a significant predictor of vaccine acceptance for healthcare students [26, 31]. Regarding age, younger age (<40) and older age (>45) were both a barrier [20, 22, 25, 36, 45] and a facilitator [5, 20–23, 25, 28–30, 32–34, 36, 39, 42, 43, 45–48] of vaccine uptake in HCWs. One meta-analysis found that being 30 years or older was a significant predictor of vaccine acceptance in this population [28]. For healthcare students, older age was associated with acceptance [40, 45, 46]. However, a meta-analysis did not find age to predict vaccine uptake for healthcare students [26] significantly. For ethnicity, this review suggests that HCWs from White ethnic backgrounds [5, 23, 34, 40] were more accepting of a COVID-19 inoculation than those from Black [23, 30, 40, 42, 45] or Latin ethnic backgrounds [34, 45]. Being from an Asian ethnic background was a barrier [23, 45] and facilitator [23] of vaccine uptake in HCWs. Similarly, healthcare students from a Non-Hispanic White background were more accepting of a COVID-19 vaccine than healthcare students from a Black/African American background [40]. Other individual factors found to be associated with vaccine hesitancy for both HCWs and healthcare students were lower educational attainment [23, 31, 32, 36, 45, 48], lower income [20, 36, 48], or residing in low-and middle-income countries [27, 35]. In fact, literature highlighted that in some low and middle-income countries, the vaccine coverage is less than 20% and, in the general population, evidence suggests that people hesitant to COVID-19 vaccine belong to low socioeconomic groups, or to Asian and black ethnic groups, Muslims, and Buddhists [52, 53] It is possible that disinformation, in association with some religious beliefs, could discourage people to take the COVID-19 vaccine [53]. Also, studies carried out in Islamic regions, have shown that the willingness to purchase the covid-19 vaccine could be one of the influencing factors regarding the acceptance of the vaccine. A recent study [54], carried out in Indonesia, showed that only a small percentage of the population was willing to purchase a COVID-19, even if, the overall acceptance rate of a COVID-19 vaccine varied based on its effectiveness and the related risk of adverse effects. Further studies should explore more deeply and specifically the association of these variables with the level of vaccine hesitancy and acceptance of a COVID-19 vaccine for HCWs and healthcare students. To reduce hesitancy, vaccination programs and public health campaigns should tailor messages to reflect the variation in sociodemographic characteristics and the social and cultural norms surrounding the target group. The information and message delivered to the target group should be presented in a format that the target group can relate to and access.

Another driver of hesitancy in HCWs and healthcare students was related to concerns about the safety, efficacy and potential side-effects of a COVID-19 vaccine [5, 20, 36, 40, 48]. Moreover, the rapid development of the vaccine, perceived lack of clinical trials and an expedited approval and production process led to greater hesitancy in HCWs [33, 42, 48]. For healthcare students, financial barriers to the vaccine also increased hesitancy [35]. Confidence or trust in the safety and efficacy of a vaccine and the systems that develop, manufacture and distribute vaccines is an integral part of the decision-making process surrounding vaccine behaviours not just for HCWs and healthcare students but for the general population as well [21, 55]. Restoring confidence and trust in the COVID-19 vaccine can be achieved by increasing transparency and awareness of scientific rigour throughout the development, approval and distribution processes [56]. Tailored communication strategies that disseminate information regarding the progress of COVID-19 vaccines, as well as scientific data about the safety and efficacy of the vaccines to HCWs, may increase trust, help to expedite the decision-making process and lead to faster uptake of a COVID-19 inoculation in this sub-population [57].

This review also found that an individual’s previous vaccination behaviours were associated with current vaccination intentions [5, 25, 26, 30, 39, 48]. A meta-analysis found that previous history of influenza vaccine uptake was a significant determinant of vaccine acceptance in HCWs [28]. These findings highlight the importance of continuously promoting favourable behaviours and attitudes toward vaccination programmes throughout an individual’s healthcare career. Vaccine promotion campaigns continuously rolled out within healthcare institutions could also play a critical role in shaping future vaccination intentions and facilitate HCWs’ willingness to vaccinate without delay in response to future infectious disease outbreaks [58]. Another driver of vaccine intentions was a history of a COVID-19 infection. HCWs and healthcare students who had already contracted COVID-19 were more hesitant to accept vaccination [26, 34, 45, 47, 48]. More than likely due to the belief that natural immunity offers a level of protection against further infections, reducing the perceived need for an inoculation [59]. However, research has found that around a third of individuals do not develop natural immunity after recovering from a COVID-19 infection [60], and in those that do, natural immunity may wane quicker than vaccine-induced immunity [61]. Similarly, the risk of re-infection is increased [62] and the chances of long-term damage (long-COVID) also increase in unvaccinated individuals [63]. Educating HCWs and healthcare students about the increased risk of re-infection due to occupational exposure and the negative health impacts of repeated exposure may be an effective strategy to encourage uptake in previously hesitant HCWs.

Several drivers related to workplace settings were also found to affect vaccination behaviours. For example, being involved in COVID-19-related duties [29, 41, 48], being in regular contact with COVID-19 infected patients [25, 34, 39, 47], perceiving a lack of PPE and inadequacies in workplace infection control policies [46] were all factors found to increase uptake behaviours in HCWs. Meta-analyses found that perceived risk of COVID-19 infection was a significant determinant of vaccine intentions for HCWs and healthcare students [26, 29]. Specifically, individuals perceiving themselves as at higher risk of contracting a COVID-19 infection were more willing to accept a vaccine [26, 29]. Negative perceptions of risk may be due to a perceived lack of likelihood, susceptibility and severity of infection within the workplace [64]. Strategies to improve uptake in HCWs have focused on implementing mandatory vaccinations; however, this strategy may be counterproductive for some individuals. For example, this review found that HCWs and healthcare students who disagreed with compulsory vaccination were more hesitant than those supporting mandatory policies [26, 31, 33, 36]. A meta-analysis found disagreement with compulsory vaccination to be a significant predictor of hesitancy in healthcare students [26]. Mandatory vaccination policies may not be the most effective approach to reduce hesitancy in this population and implementing such enforcements could have additional consequences on staffing levels and care provisions in some already-stretched medical institutions [65]. Alternative strategies focused on building trust and addressing the concerns of hesitant HCWs and healthcare students may yield greater results.

Moreover, an individual’s social network was instrumental in promoting vaccine acceptance, and this may be a potential avenue to model intervention strategies. For example, HCWs that received encouragement or recommendations to vaccinate from their close social networks, colleagues, or other healthcare professionals were more willing to accept an inoculation [29, 33, 36, 48]. Moreover, HCWs who displayed higher levels of altruism [32, 45, 48] and perceived vaccination as a collective responsibility [22, 39, 48] were more likely to accept a COVID-19 vaccine. Previous research has found that influence and pressure from social networks can be instrumental in changing behaviour [66]. For example, when close social networks engage in preventative measures (e.g. social distancing, face-mask wearing) [67] and adherence to rules is a norm endorsed by the social network [68, 69], then individuals are more likely to follow and engage in the behaviours exhibited by the social group. Therefore, strategies encouraging vaccinated HCWs and family members of HCWs to advocate for adherence to vaccination recommendations may apply social pressure and, as a result, coax hesitant HCWs into accepting a COVID-19 inoculation. Moreover, occupational communication and incentives could focus on lauding vaccinated individuals for their altruistic and collectivistic values, which may motivate hesitant HCWs to receive COVID-19 vaccination to be perceived as contributing to society’s collective behaviours.

Perceptions of the availability and adequacy of COVID-19 information and data and the sources used to seek out COVID-19-related information were found to be drivers of vaccination intentions for HCWs and healthcare students. For example, individuals who perceived a lack of adequate scientific information about the vaccine and its side effects were more hesitant to accept a COVID-19 inoculation [20, 24, 36, 48], as were those whose primary source of COVID-19 information came from social media platforms [26, 30]. Alternatively, HCWs and healthcare students who utilised reliable sources for COVID-19-related information [36, 39, 45] and perceived the information to be adequate [20, 24, 36, 48] were more accepting of a COVID-19 vaccine. This finding would suggest that levels of health literacy, defined as “*the degree to which individuals have the capacity to obtain*, *process and understand basic health information needed to make appropriate health decisions*” [70, pg. 6], may be lower in some HCWs and healthcare students. Interventions aimed at improving health literacy and digital health literacy may be efficacious in increasing vaccine acceptance in this population. For example, equipping an individual with skills to enhance their information-seeking behaviours will improve perceptions and adequacy of information and inform decision-making processes, potentially leading to voluntary uptake. Another driver of hesitancy for HCWs and healthcare students was exposure to misinformation or conspiracy theories on social media [5, 22, 32, 46]. Although efforts have been made to manage the spread of COVID-19 misinformation on social media platforms (e.g. fact-checking, misinformation awareness campaigns) [71], more could be done at an individual and occupational level to reduce the impact misinformation has on the vaccination behaviour of HCWs and healthcare students. One potential strategy would be to utilise pre-bunking; this is where the impacts (changes in beliefs and behaviours) of misinformation are neutralised by pre-exposure to accurate information [72]. As such, strategies to regularly expose HCWs and healthcare students to scientific, accurate information about COVID-19 and the vaccines from trusted medical bodies within the workplace may reduce the influence misinformation may have on the vaccination intentions of HCWs and healthcare students. Moreover, media literacy resources, such as “Go Viral”, an interactive game supported by the WHO that teaches individuals to identify and resist being influenced by COVID-19 misinformation [73], could be recommended to HCWs and healthcare students which could also reduce the likelihood of hesitancy towards COVID-19 vaccines as a result of exposure to misinformation on social media platforms.

Furthermore, the level of trust HCWs and healthcare students held towards key social actors, such as the government, public health authorities, health experts, vaccine manufacturers, pharmaceutical companies and scientists, was pivotal in shaping vaccination intentions. Specifically, a lack of trust in these social actors was associated with hesitancy toward accepting a COVID-19 vaccine [5, 36, 42, 45, 46, 48]. Moreover, a lack of trust in pandemic management [20, 21, 34] and health promotion strategies [36], as well as exposure to conflicting or unclear COVID-19 information by the government or public health figures [30, 48], lead to greater hesitancy for HCWs. Strategies that improve and build trust towards the government, health authorities and other influential social actors are imperative to ensure that vaccine uptake remains high in populations of HCWs and healthcare students. To enhance trust in COVID-19 vaccination, governments should strive to be transparent about vaccination strategies and maintain integrity and accountability throughout vaccine development, approval, distribution and administration. Information regarding these stages must be released in a timely, accurate and accessible manner to the general public and healthcare professionals. Moreover, health communication messages and campaigns delivered by the government or health officials must strive to be coherent, authoritative and free of ambiguity or conflicting information. These strategies would contribute to an individual’s decision-making process and foster trust and confidence in key social actors, ultimately leading to greater uptake of COVID-19 vaccinations in HCWs, healthcare students, and the general population [74].

In summary, this review has synthesised the most commonly reported factors associated with COVID-19 vaccine acceptance and hesitancy for HCWs and healthcare students. Frequent reasons associated with vaccination intentions were related to sociodemographic factors, COVID-19 exposure, perceived risk, attitudes towards mandatory vaccination, vaccination history, perceptions regarding the safety, efficacy and side-effects of the COVID-19 vaccine, perceptions about the rapid development, testing, approval and distribution of the vaccine, social pressure, altruism, collective responsibility, trust of key social actors, perceptions of pandemic management, perceived adequacy of information, usage of social media and exposure to misinformation. Although identifying these factors may provide potential areas for intervention, there are several points to consider. Firstly, most of the reviews included in this synthesis investigated prospective attitudes and behavioural intentions toward a COVID-19 vaccine, which does not always translate to actual behaviour [75, 76]. For example, research exploring the relationship between HCWs intentions and future acceptance of an influenza vaccine found that 42% of HCWs who intended to accept the vaccine failed to act on those intentions [77]. Future research exploring the factors that lead to changes in intentions is needed as these drivers may be pivotal to developing effective intervention strategies. Moreover, the evidence in this review only considers a single inoculation; however, some COVID-19 vaccines require multiple doses and booster shots. Hesitancy towards additional inoculations may hamper current vaccination progress and contribute to the circulation of the virus and the development and spread of new variants [78]. Therefore, future research should explore acceptance intentions towards multiple inoculations.

### 4.1 Limitations of the review

There are several limitations to this review. Firstly, a summary of the sociodemographic and occupational data for the populations included within a review was inconsistently reported. Regarding sociodemographic characteristics, there was a lack of summary data that described the participants in the primary studies in their review. For example, of the 31 reviews, 11 reported gender, seven reported age and only two reported ethnicities. Therefore, this review’s representativeness and generalisability are impeded due to a lack of transparency in reporting this information. Regarding the occupational data, there was variation in the occupational groups included in the primary studies. For example, most reviews did not focus on one particular healthcare role but included various roles ranging from frontline healthcare workers to hospital administrative staff and non-medical personnel. Moreover, seven reviews used the umbrella term HCWs and did not provide any breakdown of occupational roles. As a result, the generalisability and applicability of the determinants identified may not be entirely representative of the population under investigation. Secondly, the reviews reporting data on associations did not indicate the significance or effect sizes of the associations between the determinants and vaccine hesitancy or acceptance found in the primary studies. There was also no transparency around the factors found to be non-significant. The selective reporting within the reviews may have introduced bias surrounding the determinants reported in this synthesis, limiting our interpretation. Also, the correlational design of the primary studies impedes this review’s ability to draw any inferences regarding cause or effect. Although the results from the overlapping assessment were considered low, the impact this may have had on the amount of evidence provided for each determinant and the conclusions drawn in this review cannot be discounted. Moreover, this synthesis also included other types of reviews (e.g. scoping reviews), which could be argued to have reduced the quality of evidence and implications of this synthesis [17]. However, the methodological appraisal found that five out of six scoping reviews were of ‘moderate’ or ‘strong quality’ and were more methodologically rigorous than some of the included systematic reviews. Finally, there is a possibility that the search strategy may have missed eligible reviews. Although steps were taken to minimise this risk (i.e. consultations with an expert librarian and updated searches), selection bias is still possible. Future research should consider more specific search strategies so as to focus on specific occupational roles and determinants and perform a meta-analysis to provide a quantitative synthesis.

## 5. Conclusion

Individual decision-making regarding vaccination is a complex process, driven by a mix of scientific, social, behavioural, cultural, emotional, environmental and psychological factors. HCWs and healthcare students are key populations to consider when planning vaccination campaigns. The determinants of vaccination hesitancy among HCWs are mainly related to concerns about the vaccine’s side effects or lack of scientific information about the vaccine itself along with being affected by chronic conditions or not. Among healthcare students, non-medical students showed higher hesitancy, while students living with relatives in vulnerable conditions are less hesitant. In both populations, the lack of scientific information or the social media usage is related to vaccination hesitancy.

Strategies to reduce COVID-19 vaccine hesitancy in this population require a multifaceted approach targeting amenable drivers at the individual, occupational and societal level. At the individual level, educational interventions aimed at improving health literacy, knowledge of COVID-19 and associated vaccines, reducing susceptibility to misinformation and promoting pro-vaccination behaviours during medical training might increase COVID-19 vaccination uptake in both HCWs and healthcare students. At the occupational level, strategies to minimise vaccine hesitancy in this population may consist of distributing and ensuring access to accurate scientific information, promoting and rewarding pro-vaccination behaviours, implementing mandatory training sessions to improve health literacy, recommending engagement with media literacy resources, as well as facilitating regular interactive sessions lead by pro-vaccination HCWs to educate and address the vaccine-related concerns held by hesitant HCWs and healthcare students. At the societal level, hesitancy could be addressed through the delivery of sociodemographic-specific vaccination programs and public health campaigns, the dissemination of scientific information regarding COVID-19 vaccine progress reports to medical institutions and staff, through transparent and accurate communication messages from government and other trusted social actors, avoidance of unclear or conflicting messages to HCWs, as well as social pressure from other HCWs and broader social networks. There is evidence to suggest that these interventions may be effective at improving COVID-19 vaccine uptake; however, further research is needed to assess the efficacy of these strategies with HCWs and healthcare students.

## Supporting information

S1 Checklist PRISMA 2020 checklist. (DOCX)

S1 Table Critical appraisal results for included reviews using the JBI critical appraisal checklist for systematic reviews and research syntheses. (DOCX)

S2 Table Results from meta-analyses exploring sociodemographic characteristics as determinants of vaccine acceptance in HCWs. (DOCX)

S3 Table Results from meta-analyses exploring sociodemographic characteristics as determinants of vaccine acceptance or hesitancy in healthcare students. (DOCX)

S1 Data (XLSX)

S2 Data Overlap spreadsheet. Assessment of overlap between studies.
(XLSX)
