# Supplementary material for: Understanding the barriers and facilitators of vaccine hesitancy towards the COVID-19 vaccine in healthcare workers and healthcare students worldwide: An Umbrella Review
Source: PLoS One. 2023 Apr 12;18(4):e0280439. doi: 10.1371/journal.pone.0280439 (PMC10096263; doi:10.1371/journal.pone.0280439)
Supplement: S1 Table — (DOCX) [file pone.0280439.s002.docx]

**Supplementary Table 1.** Critical appraisal results for included reviews using the JBI Critical Appraisal Checklist for Systematic Reviews and Research Syntheses.

| No. | Author, year [Reference] | Q1 | Q2 | Q3 | Q4 | Q5 | Q6 | Q7 | Q8 | Q9 | Q10 | Q11 | Score | Quality appraisal |
| --- | --- | --- | --- | --- | --- | --- | --- | --- | --- | --- | --- | --- | --- | --- |
| 1 | Ackah et al., 2021 [24] | Y | Y | Y | Y | Y | Y | Y | Y | Y | Y | Y | 22 | Strong quality |
| 2 | Al-Amer et al., 2021 [32] | Y | Y | Y | Y | Y | Y | Y | Y | N | Y | Y | 20 | Strong quality |
| 3 | AlShurman et al., 2021 [46] | Y | Y | Y | Y | N/A | N/A | U | Y | N/A | Y | Y | 13 | Strong quality |
| 4 | Biswas et al., 2021 [5] | Y | N | N | N | N/A | N/A | N | Y | N/A | N | N | -8 | Very low quality |
| 5 | Caiazzo & Stimpfel, 2022 [23] | Y | U | Y | Y | Y | U | U | Y | N | N/A | U | 4 | Low quality |
| 6 | Dadras et al., 2022 [33] | Y | Y | Y | Y | U | U | Y | Y | N | Y | Y | 12 | Strong quality |
| 7 | Fattah et al., 2022 [41] | Y | U | Y | Y | N | N | N | N | N | N | N | -9 | Very low quality |
| 8 | Galanis et al., 2021 [25] | Y | Y | Y | Y | Y | Y | U | Y | Y | U | Y | 16 | Strong quality |
| 9 | Geng et al., 2022 [26] | Y | Y | Y | Y | Y | U | Y | Y | Y | Y | U | 16 | Strong quality |
| 10 | Hajure et al., 2021 [34] | Y | N | Y | Y | Y | U | Y | Y | N | N | N | 3 | Low quality |
| 11 | Joshi et al., 2021 [47] | Y | Y | Y | N | N/A | N/A | Y | Y | N/A | Y | Y | 12 | Strong quality |
| 12 | Khubchandani et al., 2022 [48] | Y | U | Y | Y | N/A | N/A | U | Y | N/A | Y | U | 7 | Moderate quality |
| 13 | Li et al., 2021 [42] | Y | Y | Y | Y | Y | U | Y | Y | N | Y | Y | 15 | Strong quality |
| 14 | Lin, Lee et al., 2022 [27] | Y | Y | Y | Y | Y | Y | Y | Y | Y | U | Y | 19 | Strong quality |
| 15 | Lin, Tu et al., 2021 [43] | Y | Y | Y | Y | N | U | Y | Y | N | Y | Y | 11 | Strong quality |
| 16 | Luo et al., 2021 [28] | Y | Y | Y | Y | Y | Y | U | Y | Y | U | U | 13 | Strong quality |
| 17 | Machado et al., 2021 [49] | Y | Y | Y | Y | N/A | N/A | U | Y | N/A | Y | Y | 13 | Strong quality |
| 18 | Ngangue et al., 2022 [20] | Y | Y | Y | Y | Y | Y | Y | Y | N | U | Y | 15 | Strong quality |
| 19 | Olu-Abiodun et al., 2022 [44] | Y | Y | N | Y | U | U | U | Y | N | Y | Y | 5 | Moderate quality |
| 20 | Patwary et al., 2022 [29] | Y | Y | Y | Y | Y | U | Y | Y | Y | Y | Y | 19 | Strong quality |
| 21 | Pekcan et al., 2021 [30] | Y | Y | Y | N | Y | N | N | Y | N | Y | Y | 6 | Moderate quality |
| 22 | Salomoni et al., 2021 [35] | Y | Y | Y | N | U | U | U | Y | N | Y | Y | 5 | Moderate quality |
| 23 | Shakeel et al., 2022 [36] | Y | Y | N | N | N | N | N | Y | N | Y | Y | -2 | Very low quality |
| 24 | Snehota et al., 2021 [37] | Y | Y | Y | Y | U | U | U | Y | N | U | U | 3 | Low quality |
| 25 | Troiano & Nardi, 2021 [38] | Y | N | N | N | U | U | U | Y | N | U | N | -10 | Very low quality |
| 26 | Ulbrichtova et al., 2022 [31] | Y | Y | Y | Y | Y | Y | Y | Y | Y | Y | U | 19 | Strong quality |
| 27 | Wake, 2021 [21] | Y | U | U | Y | Y | U | U | Y | N | Y | U | 3 | Low quality |
| 28 | Wake, 2021 [39] | Y | U | U | Y | Y | U | U | Y | N | Y | Y | 6 | Moderate quality |
| 29 | Wang & Liu, 2022 [45] | Y | Y | Y | N | U | U | U | Y | N | Y | Y | 5 | Moderate quality |
| 30 | Willems et al., 2021 [22] | Y | Y | Y | Y | U | U | U | Y | N/A | U | Y | 8 | Moderate quality |
| 31 | Yasmin et al., 2021 [40] | Y | Y | U | Y | U | U | U | U | N | Y | Y | 3 | Low quality |

*Note.* Y = Yes; N = No; U = Unclear; N/A = Not applicable. The 4-item response scale was scored as follows: ‘*yes*’ = 2 points, ‘*no*’ = -2 points, ‘*unclear*’ = -1 point and ‘*not applicable*’ = 0 points. Scoring interpretation: Strong quality = score: ≥10; Moderate quality = score: 5-9; Low quality = score: 0-4; Very low quality = score: ≤0.
