# Supplementary material for: Understanding the barriers and facilitators of vaccine hesitancy towards the COVID-19 vaccine in healthcare workers and healthcare students worldwide: An Umbrella Review
Source: PLoS One. 2023 Apr 12;18(4):e0280439. doi: 10.1371/journal.pone.0280439 (PMC10096263; doi:10.1371/journal.pone.0280439)
Supplement: S2 Table — (DOCX) [file pone.0280439.s003.docx]

**Supplementary Table 2.** Results from meta-analyses exploring sociodemographic characteristics as determinants of vaccine acceptance in HCWs.

| Author, Year [Reference] | Outcome | No. of individual studies included in meta-analyses | Odds ratio (Lower limit - Upper limit) | Model: I^2^ (p-value) |
| --- | --- | --- | --- | --- |
| Luo et al., 2021 [28] | Age | 4 | **1.32 (1.16-1.51)***** | FE: 31.7% (.222) |
|  | Gender | 3 | **1.99 (1.65–2.41)***** | FE: 0.0% (.369) |
|  | Occupation | 7 | 1.72 (1.25–2.38) | RE: 85.5% (.000) |
|  | History of prior influenza vaccination | 3 | **2.97 (1.82–4.84)***** | RE: 88.1% (.000) |
| Patwary et al., 2022 [29] | Female | 17 | 1 |  |
|  | Male | 17 | **1.2 (1–1.6)*** | 91.6% |
|  | Rural | 6 | 1 |  |
|  | Urban | 6 | 1 (0.6–1.5) | 92.7 |
|  | Single | 8 | 1 |  |
|  | Married | 8 | 1.2 (0.8–1.6) | 51.4% |
|  | Separated | 2 | 1.7 (0.3–9.8) | 0% |
|  | Undergraduate | 14 | 1 |  |
|  | Postgraduate | 7 | 1.5 (0.9–2.4) | 91.7% |
|  | Secondary | 13 | 1.2 (0.8–2) | 90.6% |
|  | Primary | 7 | 0.6 (0.3–1.2) | 84.7% |
|  | None | 3 | 1.4 (0.6–3) | 77.3% |
|  | Government employee | 5 | 1 |  |
|  | Private employee | 4 | 1 (0.8–1.9) | 0% |
|  | Self-employed | 4 | 0.8 (0.7–1.1) | 31.2% |
|  | Unemployed | 3 | 0.4 (0.2–1) | 93.3% |
|  | Student | 4 | 1.8 (0.9–3.5) | 88.01% |
|  | Other | 3 | 0.5 (0.1–2) | 86.8% |
|  | Chronic disease - No | 5 | 1 |  |
|  | Chronic disease – Yes | 5 | 1 (0.8–1.3) | 86.4% |
|  | Healthcare workers - No | 5 | 1 |  |
|  | Healthcare workers – Yes | 5 | 1.1 (0.7–1.8) | 94.4% |
|  | Previous vaccination - No | 3 | 1 |  |
|  | Previous vaccination - Yes | 3 | 0.9 (0.4–2.4) | 88.4% |
|  | Perceived risk - No | 3 | 1 |  |
|  | Perceived risk - Yes | 3 | **2.4 (1.1–5.5)** | 93.1% |

*Note.* FE = Fixed-effects model; RE = Random-effects model. Significant p-values are indicated in bold. *** = <.001; ** = <.01; * = <.05
