# Supplementary material for: Understanding the barriers and facilitators of vaccine hesitancy towards the COVID-19 vaccine in healthcare workers and healthcare students worldwide: An Umbrella Review
Source: PLoS One. 2023 Apr 12;18(4):e0280439. doi: 10.1371/journal.pone.0280439 (PMC10096263; doi:10.1371/journal.pone.0280439)
Supplement: S3 Table — (DOCX) [file pone.0280439.s004.docx]

**Supplementary Table 3.** Results from meta-analyses exploring sociodemographic characteristics as determinants of vaccine acceptance or hesitancy in healthcare students.

| Author, Year [Reference] | Outcome | No. of individual studies included in meta-analyses | Total sample size | Odds ratio (Lower limit - Upper limit) | Model: I^2^ (p-value) |
| --- | --- | --- | --- | --- | --- |
| Ulbrichtova et al., 2022 [31] | Gender | 5 | 3622 | 1.038 (0.874–1.233) | FE: 73.4% (.005) |
|  | Year of Study | 4 | 2001 | 2.414 (0.754–7.729) | RE: 97.5% (.000) |
|  | Compulsory vaccination | 3 | NR | **0.714 (0.670–0.754)*** | RE: 78.7% (.009) |
| Geng et al., 2022 [26] | Age | 12 | NR | 1.10 (0.97–1.22) | RE: 89.9% (<.001) |
|  | Female | 12 | NR | 0.85 (0.69–1.02) | RE: 78.6% (<.001) |
|  | Grade (senior vs junior) | 5 | NR | 1.41 (0.95–1.87) | RE: 82.0% (<.001) |
|  | Major (medical vs non-medical) | 5 | NR | **2.75 (2.00–3.50)*** | RE: 87.9% (<.001) |
|  | Self-experience of COVID-19 infection themselves | 6 | NR | **0.49 (0.19–0.78)*** | RE: 38.2% (.167) |
|  | People on social networks getting COVID-19 infection | 6 | NR | 1.06 (0.65–1.46) | RE: 90.3% (<.001) |
|  | People on whose social network died of COVID-19 infection | 3 | NR | 0.89 (0.55–1.23) | RE: 0.0% (.604) |
|  | Knowledge about COVID-19 vaccines | 3 | NR | **1.22 (1.02–1.42)*** | RE: 71.7% (.007) |
|  | Higher exposure risk of COVID-19 | 7 | NR | 1.16 (0.99–1.33) | RE: 97.8% (<.001) |
|  | Concerns about getting infected with COVID-19 | 4 | NR | **1.41 (1.05–1.78)*** | RE: 90.1% (<.001) |
|  | Importance of COVID-19 vaccination for individuals | 4 | NR | **1.24 (1.14–1.33)*** | RE: 0.0% (.870) |
|  | Vaccination is better than none | 3 | NR | 0.88 (0.07–1.68) | RE: 95.8% (<.001) |
|  | Trust in vaccine information from medical experts | 4 | NR | 2.29 (0.75–3.83) | RE: 35.9% (.197) |
|  | Concerns about the adverse effects of COVID-19 vaccines | 6 | NR | **0.57 (0.40–0.73)*** | RE: 90.7% (<.001) |
|  | Support the compulsory COVID-19 vaccination of the public | 3 | NR | **2.33 (1.13–3.53)*** | RE: 42.2% (.177) |
|  | Getting vaccinated if it compulsory | 3 | NR | **0.32 (-0.06–0.69)*** | RE: 88.8% (<.001) |
|  | Have other vaccination in the past five years | 9 | NR | 1.08 (0.82–1.34) | RE: 46.7% (.037) |

*Note.* FE = Fixed-effects model; RE = Random-effects model. Significant p-values are indicated in bold. *** = <.001; ** = <.01; * = <.05
